# Supplementary material for: ‘What does that mean?’: a qualitative exploration of the primary and secondary clinical care experiences of young people with continence problems in the UK
Source: BMJ Open. 2017 Oct 16;7(10):e015544. doi: 10.1136/bmjopen-2016-015544 (PMC5652505; doi:10.1136/bmjopen-2016-015544)
Supplement: Author's manuscript [file bmjopen-2016-015544.draft_revisions.pdf]

BMJ Open is committed to open peer review. As part of this commitment we make the peer review history of every article we publish publicly available.

When an article is published we post the peer reviewers' comments and the authors' responses online. We also post the versions of the paper that were used during peer review. These are the versions that the peer review comments apply to.

The versions of the paper that follow are the versions that were submitted during the peer review process. They are not the versions of record or the final published versions. They should not be cited or distributed as the published version of this manuscript.

BMJ Open is an open access journal and the full, final, typeset and author-corrected version of record of the manuscript is available on our site with no access controls, subscription charges or pay-per-view fees (http://bmjopen.bmj.com).

If you have any questions on BMJ Open's open peer review process please email editorial.bmjopen@bmj.com

BMJ Open

‘What does that mean?’: Clinical care experiences of young people with continence problems

Journal:	*BMJ Open*
Manuscript ID	bmjopen-2016-015544
Article Type:	Research
Date Submitted by the Author:	12-Dec-2016
Complete List of Authors:	Whale, Katie; University of Bristol, Centre for Child and Adolescent Health<br>Cramer, Helen; Centre for Academic Primary Care, School of Social and Community Medicine<br>Wright, Anne; Evelina London Children's Hospital<br>Sanders, Caroline ; University of Northern British Columbia<br>Joinson, Carol; University of Bristol, Centre for Child and Adolescent Health; School of Social and Community Medicine, University of Bristol
**Primary Subject Heading**:	Urology
Secondary Subject Heading:	Paediatrics, Qualitative research
Keywords:	Urinary incontinences < UROLOGY, Fecal incontinence, PAEDIATRICS

SCHOLARONE™ Manuscripts

For peer review only

'What does that mean?': Clinical care experiences of young people with continence problems

Whale K[1], Cramer H[2], Wright, A[3], Sanders, C[4] & Joinson C[1]

[1] Centre for Child and Adolescent Health, School of Social and Community Medicine, University of Bristol

[2] Centre for Academic Primary Care, School of Social and Community Medicine, University of Bristol

[3] Evelina London Children's Hospital, Guy's and St Thomas' NHS Foundation Trust, London

[4] University of Northern British Columbia, Canada

Corresponding author

Dr Katie Whale

Centre for Child and Adolescent Health

School of Social and Community Medicine

University of Bristol

Oakfield House

Oakfield Grove

Bristol

BS8 2BN

Email: Katie.whale@bristol.ac.uk

Phone: 0117 3313328

For peer review only

Abstract

Objectives: To explore the clinical care experiences of young people with continence problems.

Design: In-depth semi-structured qualitative interviews were conducted by Skype and telephone, with the addition of art-based participatory research techniques. Transcripts were analysed using inductive thematic analysis.

Setting: Primary and secondary care.

Participants: We interviewed 20 participants (nine females, 11 males) aged 11-20. There were six participants with bedwetting alone, five with daytime wetting alone, five with combined (day and night) wetting, and four with soiling.

Results: We identified four themes: appointment experiences, treatment experiences, engagement with treatment, and internalisation and externalisation of the continence problem. Patient-focuses appointments using age-appropriate language were highly desirable. Continuity of care was highlighted as an important aspect of positive clinical experiences, however this was found to be rare with many participants seeing a different person on each visit. Participants had tried a wide range of treatments for their continence problems with varying degrees of success. Relapse and treatment failure were common. Experiencing relapse was distressing and diminished participants' belief in the success of future treatments, and undermined adherence. Participants would be seen to adopt two opposing coping strategies for dealing with their continence problem, internalisation and externalisation.

Conclusion: Incontinence in young people is challenging to manage. Young people may need to try a range of treatments before their symptoms improve. Due to challenges in treatment there is an increased risk of poor adherence. During appointments clinicians should work to build rapport with patients and use age-appropriate language. Involving young people in their own care decisions is important. The way in which young people understand their continence problem can influence their coping strategies and adherence to treatment regimes.

For peer review only

Strengths and limitations of the study

- In-depth qualitative interviews provide a unique insight into the experiences of young people with incontinence
- Due to the sensitive nature of this topic, interviews were combined with an arts-based participatory approach to facilitate greater disclosure and gain a better insight into young people's lives
- Skype and telephone interviews allowed access to participants from a wide geographical area
- Telephone interviews presented challenges in building rapport and body language with the participants, which may have hindered some personal disclosure

This research was funded by a grant from the Medical Research Council (Increasing understanding of risk factors and outcomes associated with continence problems in children and adolescents. MRC reference: MR/L007231/1).

We have read and understood BMJ policy on declaration of interests and declare that we have no competing interests

Author contributions

Katie Whale: Lead researcher, design of qualitative work, conducting interviews, analysis of interview data, writing and preparing manuscript

Helen Cramer: Design of qualitative work, analysis of a sub-set of interviews and input into analysis process, member of the project steering group, revising manuscript critically for important intellectual content

Anne Wright: Recruitment of participants, contribution to interview design, contribution to interpretation of themes, member of the project steering group, revising manuscript critically for important intellectual content

For peer review only

Caroline Sanders: Contribution to interpretation of themes, revising manuscript critically for important intellectual content

Carol Joinson: Principal investigator, obtaining ethical approval for the study, design of qualitative work, analysis of a sub-set of interviews and input into analysis process, revising manuscript critically for important intellectual content

For peer review only

INTRODUCTION

Continence problems (daytime wetting, bedwetting, and soiling) [1] are among the most common chronic conditions of childhood. In the UK it is estimated that 900,000 children and young people experience some form of continence problem[2]. This is, however, likely to be an underestimate as the stigma associated with incontinence often prevents individuals from seeking treatment[3, 4]. Epidemiological studies estimate the prevalence of incontinence in 11–20 year olds to be around 2–3% for daytime wetting, 2–2.5% for bedwetting and 1–1.5% for soiling[5, 6]. Incontinence in children and young people has been linked to high rates of emotional distress, poor self-image, relationship problems and unhappiness at school[7-12]. Poor management of continence problems has an adverse impact on quality of life and self-esteem during childhood and adolescence[13].

A minority of children and young people have an organic (neurological, structural or anatomic) cause for their incontinence, but the vast majority of paediatric continence problems are functional (no underlying organic cause)[14, 15, 1]. Uncertainty surrounding individual responses to treatments for functional incontinence and varying levels of access to paediatric continence services are major factors affecting patients with incontinence[2]. Treatment for functional incontinence is varied and there is often no singularly effective treatment. As a consequence, patients may need to try a series of different approaches to manage or improve their incontinence symptoms[16, 17]. The first-line treatment for daytime wetting is often urotherapy, involving a structured regime of toileting and fluid intake, sometimes combined with medication[18-20]. Initial treatments for bedwetting include the bedwetting alarm and/or medication (e.g. desmopressin)[16, 21, 14]. Constipation is a common cause of soiling and can exacerbate urinary incontinence due to pressure on the bladder. The first stage of treatment for soiling usually involves laxatives or stool softeners and, in some cases, suppositories[22, 15, 23].

Although many children experience a natural resolution of their incontinence with age, there is evidence that those with severe incontinence are at risk of their problems persisting into adolescence [6, 24-27]. Often when curative treatment fails there is a refocusing on symptom management as the child continues to mature and enters into adolescence. Adolescence can be a particularly challenging time for the management of chronic health

For peer review only

problems and is frequently associated with lower levels of treatment adherence[28]. Due to the challenges of treatment, young people with persistent continence problems may see minimal improvements and may need to follow treatment regimes for extended periods before seeing any changes. Previous work has shown that adolescents often struggle to adhere to treatments that show few immediate benefits[28].

Little research has been published on the primary and secondary care experiences of young people with continence problems, especially with regard to their experiences of treatment and management of their continence problems. The overall aim of the project was to explore the impact of continence problems on young people and the specific focus of this paper is on their experiences of primary and secondary care.

For peer review only

METHODS

Participants were recruited through five secondary care paediatric continence clinics (four in England and one in Scotland), and through an advertisement on a paediatric continence charity website (The Children's Bowel and Bladder Charity, www.eric.org.uk).
Eligible participants were between 11 and 20 years, currently experiencing continence problems (bedwetting, daytime wetting, or soiling) or who previously experienced these problems after the age of 10, and able to speak and understand English.

Participants attending paediatric continence clinics were given a study information pack by their clinician. Those recruited through the advertisement were sent an information pack by the research team. Ethical approval was given by the National Research Ethics Service Committee South West - Central Bristol (14/SW/0059). The researcher contacted all interested participants by phone to describe the study and answer any questions.

In-depth semi-structured interviews were conducted with 20 young people between February 2015 and January 2016 (see table 1 for participant characteristics). Participants were given the option to be interviewed by telephone, Skype, or face to face (for participants within a 40 mile radius of Bristol). Among the participants, 11 were interviewed by Skype and nine by telephone. No participants were interviewed face to face (those given this option preferred to be interviewed by Skype or telephone). A topic guide was developed based on previous literature on the treatment, management and impact of continence problems in children and young people. The topic guide covered issues such as attending appointments, treatment experiences, school or work, and thoughts and feelings about their continence problem. Due to the exploratory nature of the study the interviews were semi-structured. The topic guide was used as a starting point for discussion, with flexibility to discuss novel areas introduced by the participants.

An arts-based participatory approach was used in the interviews. This approach is considered appropriate for children and young people since it provides additional narratives through which personal experiences can be explored[29]. A participant activity pack was developed for use prior to and during the interviews containing a graphic representation of each possible topic area, allowing the participants to write or draw their thoughts in

For peer review only

advance, if they wished to. Participants were sent the pack in advance of their interview and were given a verbal explanation of how it could be used during the initial phone call.

All participants provided written informed consent, for ages 16 and above, or parental written consent and child assent for those below 16 years. Interviews were conducted by one researcher (KW) and lasted between 34 and 99 minutes (mean 65 minutes). Data collection and analysis were conducted in parallel after completion of the first five interviews. Early analysis was used to refine the topic guide and to further explore emerging areas of interest. Interviews were audio recorded, fully transcribed, and imported into the software package NVivo10. Inductive thematic analysis was carried out following guidelines of Braun and Clarke[30]. Following completion of the first five interviews, each transcript was read and the data were free-coded across all transcripts. A selection of three transcripts were also independently free coded by the study team (CJ and HC). Codes were discussed and compared with all members of the team in order to further refine coding and to maximise rigor[31]. An initial set of agreed codes were set up within the NVivo10 database and any new codes identified from further interviews were discussed within the team and added to the coding framework. A strong theme was identified from the data on young people's experiences of clinical care.

Sample description

In total, 20 interviews were carried out with children and young people aged 11-20 years. 17 were recruited through paediatric continence clinics and three through the ERIC advertisement. Table 1 provides a full overview of participant characteristics.

For peer review only

Table 1: Participant characteristics

Participant ID no.	Gender		Continence problem	Method of interview	Organic or non-organic continence
1	M		Night wetting	Skype	Non-organic
2	M		Day and night wetting	Telephone	Non-organic
3	F		Daytime wetting	Skype	Non-organic
4	F		Daytime wetting	Skype	Organic (kidney defect)
5	F		Day and night wetting	Skype	Non-organic
6	M		Day and night wetting	Skype	Non-organic
7	M		Night wetting	Telephone	Non-organic
8	M		Night wetting	Telephone	Non-organic
9	M		Day and night wetting	Telephone	Non-organic
10	F		Night wetting	Skype	Non-organic
11	M		Daytime wetting	Skype	Non-organic
12	M		Night wetting	Telephone	Non-organic
13	F		Soiling	Skype	Non-organic
14	F		Soiling	Skype	Organic (anal stenosis)
15	F		Daytime wetting	Skype	Organic (cerebral palsy)
16	F		Daytime wetting	Telephone	Non-organic
17	M		Soiling	Telephone	Non-organic
18	F		Night wetting	Telephone	Non-organic
19	M		Soiling	Telephone	Non-organic
20	M		Day and night wetting	Skype	Non-organic

RESULTS

Themes

Three themes directly linking to clinical care experiences were identified from the data: *appointment experiences*, *treatment experiences*, and *engagement with treatment*. A fourth theme was identified from data that was not directly linked to clinical experiences but was felt to have important implications for self-management and adherence; *internalisation and externalisation of the continence problem*.

Appointment experiences: what makes a good appointment?

Participants in the study had seen a range of health professionals during their treatment journey including general practitioners (GPs), community and school nurses, paediatricians, urologists, and paediatric continence specialists. Participants' first contact with health services was most commonly with a GP or a community or school nurse. Participants recruited through the paediatric continence clinics had the experience of attending a specialist service, whilst those recruited through the online advertisement had primarily seen their GP, or a small number had seen a general paediatrician.

For peer review only

For all participants, the most important factor that influenced their appointment experiences was the communication style used by the clinician, specifically, whether the clinician used patient-centred communication and age-appropriate language. Participants expressed a strong desire for the clinician to include them in the conversation and talk to them directly (rather than to their parent), and to use language that they could understand.

> *"The language they used, and everything, made it easier. It made it kind of child friendly I suppose, which is what they should be doing." – P12, male, aged 19, night wetting*

When health professionals failed to do this, participants talked about feeling confused and annoyed.

> *"I always had to ask my mum 'what does that mean? […] he didn't even explain what it was and even my mum didn't really know what it was half the time, because they say things that they think that you'll know, but you don't know. That's frustrating because it's like, I don't know what that means and they're not telling me what that means". – P14, female, age 12, soiling*

Due to the sensitive nature of continence problems, appointments often involved questions and discussions about very personal and potentially embarrassing issues. Therefore, building trust and rapport with the clinician was especially important for young people. Continuity of care was highlighted as a factor that helped build rapport and was highly desirable. Participants who reported seeing the same clinician at each appointment described how this helped them to feel more comfortable.

> *"After my second visit I got to know her a little better and I felt more comfortable every time I went". – P9, male, age 11, day and night wetting*

Continuity of care was more likely when participants had been referred to a specialist continence clinic, but was not always guaranteed. More than half of the participants described seeing a different clinician on each visit with no explanation as to why. Participants said that new clinicians were not familiar with their case history, or gave conflicting advice to previous clinicians.

For peer review only

"when we're at the hospital it's always a new person and they always say a different thing to what the other doctor said." – P1, male, age 12, night wetting

Appointments often centred on developing treatment plans to be implemented independently at home by the young person and/or parent. Participants expressed a strong desire to be included in the decision-making process, through being given a rationale for each treatment option and a degree of choice in how to use the treatment plan at home. When health professionals engaged in shared decision-making approaches, participants reported positive appointment experiences.

"I like to know what to do and why I should do it". – P8, male, age 11, night wetting

"He's just really warm and stuff and he just says 'How are you feeling? Do you want to do that? Do you not want to do that?'". – P14, female, age 12, soiling

Participants described wanting to be treated as an intelligent person and not wanting to be patronised by their clinician. Being seen as an expert in relation to themselves and their body was important.

"I like them to treat me as if I'm the person in question, because I'm not stupid, I know I'm not brilliant at medicine or anything, but I can do my research. I know about the medics of it. I know about the treatments that I had, and I know about me, more than anyone". – P2, male, age 14, day and night wetting

Treatment experiences: the frustration of no magic bullets

Participants often recalled trying a wide range of treatments including urotherapy, dietary changes, medication(s), bedwetting alarms, Botox injections into the bladder, laxatives, suppositories, TENS machine, and hypnotherapy. Almost all participants had tried more than one treatment and had experienced some form of relapse or complete treatment failure. Participants explained that with each failed treatment or relapse, their belief in the success of subsequent treatments diminished.

"For the first four months, it was fine…it was a lot better. Then it started to get worse again, and by six or seven months, it was actually worse than it was before I had the [Botox] operation. We went back to do a second one…it was fine for six months. Then

For peer review only

it got worse again, and then they called us back to have another one, and we said no. By that point, I was kind of sick of it, and I knew that it wasn't going to work". – P2, male, age 14, day and night wetting

Belief in treatments seemed to be made much more difficult because of the uncertainty of the cause of young people's continence problems. A minority of participants had been given a medical explanation for their incontinence and reported that this was helpful with respect to the treatments then offered.

"I think it made me more comfortable, knowing it was actually a problem and it wasn't just my mum being frantic or something". – P4, female, age 14, daytime wetting

The majority of participants, however, were given no medical explanation for their incontinence. Participants said that they found this frustrating and confusing.

"I really struggled, just thinking, 'Why has it become a problem for me?' Because there was not any reason". – P3, female, age 18, daytime wetting

Not being able to match suggested treatments to an underlying cause of the incontinence was also challenging for participants to understand. Other participants had been told there was nothing wrong with them.

"Him saying there's nothing wrong with me, he made me feel quite vulnerable […] it really made me feel bad because we were told that he is the man to see…if he can't do anything about it, like who can?". – P11, male, age 14, daytime wetting

Participants talked extensively about their emotional reactions to treatments saying that the lack of explanation, unpredictability of treatment success combined with experiences of relapse, and treatment failure, led them to feel upset and cynical about the chances of resolution. Many described giving up hope that their problem would ever go away.

"Some of it's obviously a little bit upsetting because I've had so many different medications and trying new things and it hasn't really worked yet, so my hope of it going to getting smaller and smaller". – P16, female, age 15, daytime wetting

For peer review only

For older participants, who had often been undergoing treatment for several years, feelings of pessimism were particularly common.

> *"For quite a lot of the time the, I felt really really pessimistic about this problem, just like there is absolutely no hope that it's ever going to be better and I'm going to be stuck with this problem forever. Clearly I don't want that to happen, so that really upsets me about it". – P3, female, age 18, daytime wetting*

Engagement with treatment: the effects of disappointing results

Participants reported varying levels of engagement with their treatment. Experiencing relapse or treatment failure led many participants to disengage from their treatment, with some admitting that they avoided taking their medication or lied about this to their parents.

> *"Instead of taking the tablets, I think mum thought I was doing it, say, once a week or something, I was [throwing them away] every day and having problems every day because of that".- P4, female, age 14, daytime wetting*

One participant talked about feeling so frustrated with the lack of improvement in her symptoms that she decided no treatment was going to work and there was no point trying anything new.

> *"I think it was because I'd been on them [the medication] for so long, I didn't feel like they were doing anything. I've never actually tried it [the new medication]. I've never actually properly tried it […] I just decided that it wouldn't work either". – P4, female, age 14, daytime wetting*

Another participant described disengaging from treatment not due to lack of belief in its efficacy, but as an effort to regain her independence.

> *"I think there was a period when I was in year nine [aged 13-14] where I didn't take my medication very well and it just got worse. […] I was just like 'I can do it on my own, I'm an independent woman, I can do what I want". – P15, female, age 14, daytime wetting*

For peer review only

Internalisation and externalisation of the continence problem

Through discussion with participants about their beliefs and attitudes towards their continence problem, two contrasting coping mechanisms were identified; internalisation and externalisation of the continence problem. Participants who internalised their continence problem integrated it into their self-identity. They viewed the problem as a part of their lives and themselves that needed to be actively managed, but not as something that defined or controlled them. Participants who demonstrated internalisation talked about a process of accepting that they had a continence problem, and the need to actively manage it. Taking ownership of their continence problem and embracing it because it made them 'different' and 'unique' were common lines of thought. Others talked about how this attitude helped them to cope with worries about other people finding out about their problem.

> *"I kind of realised that, actually, it's something I can't change, and it's a part of me, so I shouldn't be scared of people knowing. It's not something I've done wrong" – P2, male, age 14, daytime and night wetting*

In contrast, those who externalised their continence problem saw it as something separate to themselves and their identity, and as an intruder into their lives. Participants who demonstrated externalisation often avoided thinking about their continence problem and talked about distancing themselves from their problem and rejecting it from their self-identity.

> *"…it's just frustrating also because you know what's going to happen. So you kind of always, that's always at the back of your mind. So I do then just try and avoid it [thinking about it] if I can" – P12, male, age 19, night wetting*

In order to deal with stressful social situations, such as sleepovers or school trips, participants said they tried to convince themselves it would just be fine, even if they knew this was highly unlikely.

> *"I think when you have this issue you're trying to put aside, you know the problems but you're trying to get it out of your head, so you will do all you can to convince yourself that you'll be fine even if you won't be". – P11, male, age 14, night wetting*

For peer review only

DISCUSSION

Appointment experiences

Through interviews with young people with continence problems, we find that positive treatment experiences are characterised by seeing the same clinician at each appointment and by the use of age-appropriate communication. Building a relationship with the same clinician was desirable as it facilitated greater trust and rapport and supported young people to feel more comfortable disclosing highly personal information. Participants expressed a strong desire to be given a full explanation of their continence problem, appropriate to their age and level of understanding. This finding is consistent with studies of clinical experiences of young people with other chronic health problems[32-35]. Young people in this study wanted to be involved in making decisions about their treatment options, rather than the traditional focus on parent-doctor communication in paediatric consultations. Aligned to this, it is now recommended that children and young people should be the primary focus of their own appointments[36, 16]. There is evidence that this facilitates better communication and patient understanding, supports patient autonomy and competence, and promotes successful behaviour change and illness management[37, 38]. Shared decision making between clinician and patient could aid treatment engagement and autonomy in young people and provide them with skills and confidence to transition into adult care.

Factors affecting treatment engagement

The majority of participants in this study had functional incontinence. In the absence of an underlying organic cause of the incontinence, clinicians were often unable to give specific guidance on treatments and prognosis, creating significant illness uncertainty for the participants[39, 40]. This presents challenges since participants often had to try a range of treatments, with varying success[1]. Treatment failures and relapses were common and undermined participants' beliefs in future treatment success and the controllability of their continence problem.

Illness uncertainty and lack belief in the controllability of a health problem are significant barriers to adherence and engagement, because individuals are less likely to follow a

treatment regime they do not believe will work[41, 42]. Models of health behaviour change consistently highlight the perceived controllability of a health problem as a key factor in predicting health behaviours and treatment adherence[43-44]. Additionally, illness uncertainty has been linked to maladaptive coping, increased psychological distress, depression, and reduced quality of life[45]. When illness uncertainty is an issue, it is crucial that clinicians work to positively engage patients and support them in long-term self-management in order to minimise the impact of their health problem on daily life. Successful self-management also promotes great individual autonomy and can help patients to feel more in control of their bodies[46]. Young people with functional incontinence could also benefit from psychological support and interventions to manage uncertainty and reduce anxiety, such as acceptance commitment therapy[47]. Finding a balance between giving patients hope and being realistic about treatment success is vital in managing patient expectations, and could help to mitigate against negative treatment experiences. By increasing understanding of how treatment failures and relapses affect patients, health professionals can identify potential periods of high-risk non-adherence and take steps to manage this.

Internalisation and externalisation of the problem

Amongst the participants in our sample there was a dichotomy between those who demonstrated internalisation and externalisation of their continence problem. Those who internalised their continence problem had more successfully incorporated this into their self-identity. They recognised it as an ongoing part of their lives, but did not feel restrained or defined by it. In contrast, those who externalised their problem saw it as an external factor that intruded into their life and avoidance coping was common in this group.

Although participants did not directly link these coping strategies to their clinical experiences, the way in which they view their health condition does have significant implications for self-management and adherence to treatment. By viewing continence problems as external to the self, this undermines beliefs in the controllability of the condition, which are linked to treatment adherence[43, 44, 48]. If an individual engages in avoidance coping, they are likely to avoid any stimulus that reminds them of their continence problem, including treatment regimes. Non-adherence increases the risk of treatment failure or relapses, which may further reinforce the belief that the condition is

For peer review only

uncontrollable, creating a self-perpetuating cycle of disengagement and treatment failure. In contrast, acceptance of a chronic health problem and integration of illness experiences where the condition is viewed as only one characteristic of an individual's life are linked to a greater sense personal control and proactive management behaviours[49-51].

Whilst within our sample internalisation and externalisation is seen as two different groups, it is likely that within the wider population these responses fall along a continuum. Additionally, individuals may be likely to switch between the two attitudes depending on their experiences. Experiencing relapse or feeling overwhelmed by the condition may promote more externalising attitudes in order to cope. Alternatively, successful treatment and management symptoms may support greater internalisation. Without more exploration of these issues, the exact relationships and mechanism at work are not clear, and further work is needed to better understand this.

LIMITATIONS

Only a small number of participants with soiling were recruited into this study. Young people with soiling problems reported some different experiences to those with urinary incontinence, suggesting that further exploration is needed. Clinicians involved in recruiting to the study reported that young people with soiling were generally less engaged during appointments and would often not admit to having a problem. This led to problem recruiting these individuals. The participants with soiling either had a medical explanation for their problem (n=1), or were experiencing significant improvements in their symptoms (n=3). Another limitation of the sample was that all the participants in this study were receiving medical help for their continence problem and the majority were seeing specialists. Young people who have not received specialist help for their continence problems are likely to have different experiences to the participants in this study. This study could not explore cultural differences in young people's experiences of continence problems since all but one of the participants identified as white British. Socioeconomic background could also impact on experiences of young people with continence problems, but this data was not collected in the current study.

For peer review only

CONCLUSIONS AND FUTURE DIRECTIONS

Incontinence is challenging to treat and manage, particularly when there is no organic cause. Long-term self-management and helping individuals regain a sense of control is key in minimising the impact of incontinence on daily life. Ensuring positive appointment experiences and engaging young people in their own healthcare is vital. Clinicians should use age-appropriate language and work to build rapport in order to promote trust and disclosure. Relapses or treatment failure are common and can have a negative emotional impact and undermine beliefs in future treatment success. Assessment of coping styles may a beneficial addition to clinical assessment. For patients who are disengaged or have low treatment adherence, providing psychological support to promote acceptance may be an important first step in successful long-term self-management.

For peer review only

REFERENCES

1 Neveus T, von Gontard A, Hoebeke P, et al. The standardisation of terminology of lower urinary tract function in children and adolescents: Report from the standardisation committee of the International Children's Continence Society. *The Journal of Urology,* 2006;176: 314-324

2 Paediatrinc Continence Forum. Paediatric Continence Commissioning Guide: A handbook for the commissioning and running of paediatric continence services. 2014.

3 NICE. Assumptions used in estimating a population benchmark. 2015. Available: https://www.nice.org.uk/proxy/?sourceurl=http://www.nice.org.uk/usingguidance/commissioningguides/biologicaltherapies/assumptionsusedinestimatingapopulationbenchmark.jsp [Accessed 3 August 2016]

4 NICE. Excellence in continence care. 2015 Available: https://www.england.nhs.uk/commissioning/wp-content/uploads/sites/12/2015/11/EICC-guidance-final-document.pdf [accessed 31 August 2016)

5 Public Health England. Continence needs assessment. 2013. Available: http://atlas.chimat.org.uk/IAS/profiles/profile?profileId=45&geoTypeId= [Accessed 3 August 2016]

6 Joinson C, Grzeda M, von Gontard A, et al.(2016). The association between trajectories of bedwetting and daytime wetting in childhood and incontinence and lower urinary tract symptoms in adolescence. *Archives of Disease in Childhood 2016;* 101(S1): A187-188

7 Issenman RM, Filmer RB, Gorski PA. A review of bowel and bladder control development in children: how gastrointestinal and urologic conditions relate to problems in toilet training. *Pediatrics* 1999; 103(6 Pt 2): 1346-52.

8 Bower WF. Self-reported effect of childhood incontinence on quality of life. *J Wound Ostomy Continence Nurs* 2008;35(6):617-21.

9 Landgraf JM, Abidari J, Cilento BG, et al. Coping, commitment, and attitude: quantifying the everyday burden of enuresis on children and their families. *Pediatrics* 2004;113(2):334-344.

10 von Gontard A, Baeyens D, Van Hoecke E, et al. Psychological and psychiatric issues in urinary and fecal incontinence. *Journal of Urology* 2011;185(4):1432-6.

11 Grzeda M, Heron J, von Gontard A, Joinson C. Effects of urinary incontinence on psychosocial outcomes in adolescence. European Child & Adolescent Psychiatry. In press.

12 Filce HG & LaVergne L. Absenteeism, educational plans, and anxiety among children with incontinence and their parents, *Journal of School Health* 2015; 85(4): 241-250

13 Bower WF. Self-reported effect of childhood incontinence on quality of life, *Journal of Wound, Ostomy, and continence nursing* 2008;36(6): 617-21.

14 von Gontard A. Enuresis. In Rey JM, eds. *IACAPAP e-Textbook of Child and Adolescent Mental Health.* Geneva: International Association for Child and Adolescent Psychiatry and Allied Professions 2012a

15 von Gontard A. Encopresis. In Rey JM, eds. *IACAPAP e-Textbook of Child and Adolescent Mental Health.* Geneva: International Association for Child and Adolescent Psychiatry and Allied Professions 2012b

16 Abrams P, Anderson KE, Brubaker L, et al. Evaluation and treatment of urinary incontinence, pelvic organ prolapse and faecal incontinence, *3rd International Consultation on Incontinence: Recommendations of the International Scientific Committee.* 2005. Available: http://www.ics.org/publications/ici_3/v2.pdf/summary.pdf

17 Buckley BS, Sanders CD, Kwong JS, et al. Conservative treatment for functional daytime urinary incontinence in children. *Cochrane Database of Systematic Reviews* 2016; 9: 1-11.

18 Maternik M, Krzeminska K, Zurowska A. The management of childhood urinary incontinence. *Pediatric Nephrology* 2015;30(1):41-50.

19 Mulders MM, Cobussen-Boekhorst H, de Gier RP, et al. Urotherapy in children: quantitative measurements of daytime urinary incontinence before and after treatment according to the new definitions of the International Children's Continence Society. *Journal of Pediatric Urology* 2011;7(2):213-8.

For peer review only

20 Hagstroem S, Rittig S, Kamperis K, et al.. Timer watch assisted urotherapy in children: a randomized controlled trial. *Journal of Urology* 2010;184(4):1482-8.

21 NICE. Bedwetting in under 19s, NICE guidelines. 2010. Available at: https://www.nice.org.uk/guidance/cg111/chapter/Key-priorities-for-implementation [accessed 18/08/16]

22 Burger RE, Mugie SM, Chase J, et al. Management of functional constipation in children with lower urinary tract symptoms: report from the standardization committee of the International Children's Continence Society. *The Journal of Urology* 2013*;*190: 29-36.

23 NICE. Constipation in children and young people: diagnosis and management, Clinical Guidelines. 2010. Available at: https://www.nice.org.uk/Guidance/CG99 [accessed 19 Sept 2016]

24 Yeung CK, Sreedhar B, Sihoe JD, et al. Differences in characteristics of nighttime 337 enuresis between children and adolescents: a critical appraisal from a large 338 epidemiological study. *BJU International* 2006;97(5):1069-1073.

25 Swithinbank LV, Brookes ST, Shepherd AM et al. The natural history of urinary 349 symptoms during adolescence. *BJU* 1998;81 Suppl 3:90-3.

26 Hellström AL, Hansson E, Hansson S, et al. Micturition habits and incontinence at 351 age 17 - reinvestigation of a cohort studied at age 7. *British Journal of Urology* 1995;76: 231-234.

27 Kyrklund K, Taskinen S, Rintala RJ, et al. Lower urinary tract symptoms from 353 childhood to adulthood: a population based study of 594 Finnish individuals 4 to 26 354 years old. *Journal of Urology* 2012;188(2):588-93.

28 Taddeo D, Egedy M, Frappier JY. Adherence to treatment in adolescents. *Paediatric Child Health* 2008;13(1):19-24.

29 Carter B. & Ford K. Researching children's health experiences: The place for participatory, child-centred, arts-based approaches, *Research in Nursing and Health* 2013; 36(1): 95-107.

30 Braun V. & Clarke V. Using thematic analysis in psychology. *Qualitative Research in* Psychology 2006; 3 (2). pp. 77-101.

31 Tracey SJ. Qualitative quality: Eight "Big-Tent" criteria for excellent qualitative research, *Qualitative Inquiry* 2010;16(10): 837-851.

32 de Winter M, Baerveldt C, Kooistra J. Enabling children: Participation as a new perspective on child-health promotion. *Child Care Health Development* 2002;28:109–16.

33 Holtzheimer L, Mohay H, Masters IB. Educating young children about asthma: Comparing the effectiveness of developmentally appropriate asthma education videotape and picture book. *Child Care Health Development* 1998;24:85–99.

34 Ginsburg KR, Menapace AS, Slap GB. Factors affecting the decision to seek health care: The voice of adolescents. *Pediatrics* 1997;100:922–30.

35 Freed LH, Ellen JM, Irwin CE, et al. Determinants of adolescent satisfaction with health care providers and intentions to keep follow-up appointments. *Journal of Adolescent* Health 1998; 22:475–9.

36 Department for Health. Getting the right start: The national service framework for children, young people and maternity services – Standards for hospital services. London: Stationary Office 2008.

37 Deci EL & Ryan RM. Intrinsic Motivation and Self-Determination in Human Behaviour. New York: Plenum Publishing Co. 1985.

38 Deci EL & Ryan RM. Self-determination theory: A macrotheory of human motivation, development, and health. *Psychologie canadienne* 2008;49(3): 182-185.

39 Mishel MH. Uncertainty in illness. *Image: The Journal of Nursing Scholarship* 1988; 20(4): 225–232.

For peer review only

40 Mishel MH. Reconceptualization of the uncertainty in illness theory.Image: *Journal of Nursing Scholarship* 1990; 22(4): 256-262.

41 Ballif M, Ledergerber B, Battegay, M. Impact of previous virological treatment failures and adherence on the outcome of antitretroviral therapy in 2007, *PLoS ONE 2009;,* 4(12): e8275

42 Mann D, Ponieman D, Leventhal H, et al. Predictors of adherence to diabetes medications: The role of disease and medication beliefs. *Journal of Behavioural Medicine* 2009; 32(3): 278-284.

43 Zugelj U, Zupancic M, Komidar L, et al. Self-reported adherence beahviours in adolescent hypertensive patients: the role of illness representations and personality. *Journal of Paediatric Psychology* 2010: 1-12.

44 Leventhal H, Brissette I & Leventhal EA. The common-sense model of self-regulation of health and illness. In: Cameron LD, Leventhal H, eds. The Self-Regulation of Health and Illness behaviour. London: Routledge 2003: 42-65.

45 Wright LJ, Afari N. & Zautra, A. The illness uncertainty concept: a review. *Current Pain and Headache Reports* 2009; 13: 133-138.

46 Williams GC, McGregor HA, Zeldman A, et al. Testing a self-determination theory process model for promoting glycemic control through diabetes self-management. *Health Psychology* 2004; 23(1): 58-66.

47 Hayes SC, Luoma JB, Bond FW, et al. Acceptance and commitment therapy: model, processes and outcomes. *Behaviour Research and Therapy* 2006; 44: 1-25.

48 Leventhal H, Diefenbach M. & Leventhal E. Illness cognition: Using common sense to understand treatment adherence and affect cognition interactions. *Cognitive Therapy and Research* 1992; 16: 143-163.

49 Li L. & Moore D. Acceptance of disability and its correlates. *Journal of Social Psychology* 1998; 138: 13-25.

50 Stuifbergen A, Becker H, Blozis, S, et al. Conceptualization of development of the acceptance of chronic health conditions scale. *Issues in Mental Health Nursing* 2008;29: 101-114.

51 Karademas EC, Tsagaraki A. & Lambrou N. Illness acceptance, hospitalization stress, and subjective health in a sample of chronic patients admitted to hospital. *Journal of Health Psychology 2009;* 14:1243–1250

For peer review only

BMJ Open

‘What does that mean?’: A qualitative exploration of the clinical care experiences of young people with continence problems

Journal:	*BMJ Open*
Manuscript ID	bmjopen-2016-015544.R1
Article Type:	Research
Date Submitted by the Author:	28-Apr-2017
Complete List of Authors:	Whale, Katie; University of Bristol, Centre for Child and Adolescent Health<br>Cramer, Helen; Centre for Academic Primary Care, School of Social and Community Medicine<br>Wright, Anne; Evelina London Children's Hospital<br>Sanders, Caroline ; University of Northern British Columbia<br>Joinson, Carol; University of Bristol, Centre for Child and Adolescent Health; School of Social and Community Medicine, University of Bristol
**Primary Subject Heading**:	Urology
Secondary Subject Heading:	Paediatrics, Qualitative research
Keywords:	Urinary incontinences < UROLOGY, Fecal incontinence, PAEDIATRICS

SCHOLARONE™
Manuscripts

For peer review only

‘What does that mean?’: A qualitative exploration of the clinical care experiences of young people with continence problems

Whale K[1], Cramer H[2], Wright, A[3], Sanders, C[4] & Joinson C[1]

[1] Centre for Child and Adolescent Health, School of Social and Community Medicine, University of Bristol

[2] Centre for Academic Primary Care, School of Social and Community Medicine, University of Bristol

[3] Evelina London Children’s Hospital, Guy’s and St Thomas’ NHS Foundation Trust, London

[4] University of Northern British Columbia, Canada

Corresponding author

Dr Katie Whale

Centre for Child and Adolescent Health

School of Social and Community Medicine

University of Bristol

Oakfield House

Oakfield Grove

Bristol

BS8 2BN

Email: Katie.whale@bristol.ac.uk

Phone: 0117 3313328

Data sharing statement

Data from the study is classified as controlled access. No participant consent was given to share their interview data, therefore no further data from this study is available.

For peer review only

For peer review only - http://bmjopen.bmj.com/site/about/guidelines.xhtml

Abstract

Objectives: To explore the clinical care experiences of young people with continence problems.

Design: In-depth semi-structured qualitative interviews were conducted by Skype and telephone, with the addition of art-based participatory research techniques. Transcripts were analysed using inductive thematic analysis.

Setting: Primary and secondary care in the UK.

Participants: We interviewed 20 participants (nine females, 11 males) aged 11-20. There were six participants with bedwetting alone, five with daytime wetting alone, five with combined (day and night) wetting, and four with soiling.

Results: We identified four themes: appointment experiences, treatment experiences, engagement with treatment, and internalisation and externalisation of the continence problem. Patient-focused appointments using age-appropriate language were highly desirable. Continuity of care was highlighted as an important aspect of positive clinical experiences, however this was found to be rare with many participants seeing a different person on each visit. Participants had tried a wide range of treatments for their continence problems with varying degrees of success. Relapse and treatment failure were common. Experiencing relapse was distressing and diminished participants' belief in the success of future treatments, and undermined adherence. Participants would be seen to adopt two opposing coping strategies for dealing with their continence problem, internalisation and externalisation.

Conclusion: Incontinence in young people is challenging to manage. Young people may need to try a range of treatments before their symptoms improve. Due to challenges in treatment there is an increased risk of poor adherence. During patient-focused appointments clinicians should work to build rapport with patients and use age-appropriate language. Involving young people in their own care decisions is important. The way in which young people understand their continence problem can influence their coping strategies and adherence to treatment regimes.

For peer review only

For peer review only - http://bmjopen.bmj.com/site/about/guidelines.xhtml

Strengths and limitations of the study

- In-depth qualitative interviews provide a unique insight into the experiences of young people with incontinence
- Due to the sensitive nature of this topic, interviews were combined with an arts-based participatory approach to facilitate greater disclosure and gain a better insight into young people's lives
- Skype and telephone interviews allowed access to participants from a wide geographical area
- Telephone interviews presented challenges in building rapport and body language with the participants, which may have hindered some personal disclosure

This research was funded by a grant from the Medical Research Council (Increasing understanding of risk factors and outcomes associated with continence problems in children and adolescents. MRC reference: MR/L007231/1).

We have read and understood BMJ policy on declaration of interests and declare that we have no competing interests

Author contributions

Katie Whale: Lead researcher, design of qualitative work, conducting interviews, analysis of interview data, writing and preparing manuscript

Helen Cramer: Design of qualitative work, analysis of a sub-set of interviews and input into analysis process, member of the project steering group, revising manuscript critically for important intellectual content

Anne Wright: Recruitment of participants, contribution to interview design, contribution to interpretation of themes, member of the project steering group, revising manuscript critically for important intellectual content

For peer review only - http://bmjopen.bmj.com/site/about/guidelines.xhtml

Caroline Sanders: Contribution to interpretation of themes, revising manuscript critically for important intellectual content

Carol Joinson: Principal investigator, obtaining ethical approval for the study, design of qualitative work, analysis of a sub-set of interviews and input into analysis process, revising manuscript critically for important intellectual content

For peer review only

For peer review only - http://bmjopen.bmj.com/site/about/guidelines.xhtml

INTRODUCTION

Continence problems (daytime wetting, bedwetting, and soiling) [1] are among the most common chronic conditions of childhood. In the UK it is estimated that 900,000 children and young people experience some form of continence problem[2]. This is, however, likely to be an underestimate as the stigma associated with incontinence often prevents individuals from seeking treatment[3, 4]. Epidemiological studies estimate the prevalence of incontinence in 11–20 year olds to be around 2–3% for daytime wetting, 2–2.5% for bedwetting and 1–1.5% for soiling[5, 6]. Incontinence in children and young people has been linked to high rates of emotional distress, poor self-image, relationship problems and unhappiness at school[7-12]. Poor management of continence problems has an adverse impact on quality of life and self-esteem during childhood and adolescence[13].

A minority of children and young people have an organic (neurological, structural or anatomic) cause for their incontinence, but the vast majority of paediatric continence problems are functional (no underlying organic cause)[14, 15, 1]. Uncertainty surrounding individual responses to treatments for functional incontinence and varying levels of access to paediatric continence services are major factors affecting patients with incontinence[2]. Treatment for functional incontinence is varied and there is often no singularly effective treatment. As a consequence, patients may need to try a series of different approaches to manage or improve their incontinence symptoms[16, 17]. The first-line treatment for daytime wetting is often urotherapy, involving a structured regime of toileting and fluid intake, sometimes combined with medication[18-20]. Initial treatments for bedwetting include the bedwetting alarm and/or medication (e.g. desmopressin)[16, 21, 14]. Constipation is a common cause of soiling and can exacerbate urinary incontinence due to pressure on the bladder. The first stage of treatment for soiling usually involves laxatives or stool softeners in addition to a toileting programme and, in some cases, suppositories[22, 15, 23].

Although many children experience a natural resolution of their incontinence with age, there is evidence that those with severe incontinence are at risk of their problems persisting into adolescence [6, 24-27]. Often when curative treatment fails there is a refocusing on symptom management as the child continues to mature and enters into adolescence. Adolescence can be a particularly challenging time for the management of chronic health

For peer review only - http://bmjopen.bmj.com/site/about/guidelines.xhtml

problems and is frequently associated with lower levels of treatment adherence[28]. Due to the challenges of treatment, young people with persistent continence problems may see minimal improvements and may need to follow treatment regimes for extended periods before seeing any changes. Previous work has shown that adolescents often struggle to adhere to treatments that show few immediate benefits[28,29].

Little research has been published on the primary and secondary care experiences of young people with continence problems, especially with regard to their experiences of treatment and management of their continence problems. The overall aim of the project was to explore the impact of continence problems on young people and the specific focus of this paper is on their experiences of primary and secondary care.

For peer review only

METHODS

Participants were recruited through five secondary care paediatric continence clinics (four in England and one in Scotland), and through an advertisement on a paediatric continence charity website (The Children's Bowel and Bladder Charity, www.eric.org.uk).
Eligible participants were between 11 and 20 years, currently experiencing continence problems (bedwetting, daytime wetting, or soiling) or who previously experienced these problems after the age of 10, and able to speak and understand English.

Participants attending paediatric continence clinics were given a study information pack by their clinician. Those recruited through the advertisement were sent an information pack by the research team. Ethical approval was given by the National Research Ethics Service Committee South West - Central Bristol (14/SW/0059). The researcher contacted all interested participants by phone to describe the study and answer any questions. In total 45 potential participants were identified (36 through clinics and 9 through ERIC). 25 participants were interviewed for the following reasons: 12 unable to contact, 5 not interested in taking part, 8 withdrawn.

In-depth semi-structured interviews were conducted with 20 young people between February 2015 and January 2016 (see table 1 for participant characteristics). Participants were given the option to be interviewed by telephone, Skype, or face to face (for participants within a 40 mile radius of Bristol). Among the participants, 11 were interviewed by Skype and nine by telephone. No participants were interviewed face to face (those given this option preferred to be interviewed by Skype or telephone). A topic guide was developed based on previous literature on the treatment, management and impact of continence problems in children and young people. The topic guide covered issues such as attending appointments, treatment experiences, school or work, and thoughts and feelings about their continence problem. Due to the exploratory nature of the study the interviews were semi-structured. The topic guide was used as a starting point for discussion, with flexibility to discuss novel areas introduced by the participants.

An arts-based participatory approach was used in the interviews. This approach is considered appropriate for children and young people since it provides additional narratives

For peer review only - http://bmjopen.bmj.com/site/about/guidelines.xhtml

through which personal experiences can be explored[30]. A participant activity pack was developed for use prior to and during the interviews containing a graphic representation of each possible topic area, allowing the participants to write or draw their thoughts in advance, if they wished to. Participants were sent the pack in advance of their interview and were given a verbal explanation of how it could be used during the initial phone call.

All participants provided written informed consent, for ages 16 and above, or parental written consent and child assent for those below 16 years. Interviews were conducted by one female researcher with extensive experience of qualitative work (KW: DHealthPsy, Senior Research Associate) and lasted between 34 and 99 minutes (mean 65 minutes). Data were recorded using an encrypted audio recorder. Data collection and analysis were conducted in parallel after completion of the first five interviews. Early analysis was used to refine the topic guide and to further explore emerging areas of interest. Interviews were audio recorded, fully transcribed, and imported into the software package NVivo10. Inductive thematic analysis was carried out following guidelines of Braun and Clarke[31]. Following completion of the first five interviews, each transcript was read and the data were free-coded across all transcripts. A selection of three transcripts were also independently free coded by the study team (CJ and HC). Codes were discussed and compared with all members of the team in order to further refine coding and to maximise rigor[32]. An initial set of agreed codes were set up within the NVivo10 database and any new codes identified from further interviews were discussed within the team and added to the coding framework. A strong theme was identified from the data on young people's experiences of clinical care.

Sample description

In total, 20 interviews were carried out with children and young people aged 11-20 years. 17 were recruited through paediatric continence clinics and three through the ERIC advertisement. Table 1 provides a full overview of participant characteristics.

For peer review only - http://bmjopen.bmj.com/site/about/guidelines.xhtml

Table 1: Participant characteristics

Participant ID no.	Gender		Continence problem	Method of interview	Organic or non-organic continence
1	M		Night wetting	Skype	Non-organic
2	M		Day and night wetting	Telephone	Non-organic
3	F		Daytime wetting	Skype	Non-organic
4	F		Daytime wetting	Skype	Organic (kidney defect)
5	F		Day and night wetting	Skype	Non-organic
6	M		Day and night wetting	Skype	Non-organic
7	M		Night wetting	Telephone	Non-organic
8	M		Night wetting	Telephone	Non-organic
9	M		Day and night wetting	Telephone	Non-organic
10	F		Night wetting	Skype	Non-organic
11	M		Daytime wetting	Skype	Non-organic
12	M		Night wetting	Telephone	Non-organic
13	F		Soiling	Skype	Non-organic
14	F		Soiling	Skype	Organic (anal stenosis)
15	F		Daytime wetting	Skype	Organic (cerebral palsy)
16	F		Daytime wetting	Telephone	Non-organic
17	M		Soiling	Telephone	Non-organic
18	F		Night wetting	Telephone	Non-organic
19	M		Soiling	Telephone	Non-organic
20	M		Day and night wetting	Skype	Non-organic

For peer review only

For peer review only - http://bmjopen.bmj.com/site/about/guidelines.xhtml

RESULTS

Themes

Three themes directly linking to clinical care experiences were identified from the data: *appointment experiences*, *treatment experiences*, and *engagement with treatment*. A fourth theme was identified from data that was not directly linked to clinical experiences but was felt to have important implications for self-management and adherence; *internalisation and externalisation of the continence problem*.

Appointment experiences: what makes a good appointment?

Participants in the study had seen a range of health professionals during their treatment journey including general practitioners (GPs), community and school nurses, paediatricians, urologists, and paediatric continence specialists. Participants' first contact with health services was most commonly with a GP or a community or school nurse. Participants recruited through the paediatric continence clinics had the experience of attending a specialist service, whilst those recruited through the online advertisement had primarily seen their GP, or a small number had seen a general paediatrician.

For all participants, the most important factor that influenced their appointment experiences was the communication style used by the clinician, specifically, whether the clinician used patient-centred communication and age-appropriate language. Participants expressed a strong desire for the clinician to include them in the conversation and talk to them directly (rather than to their parent), and to use language that they could understand.

> *"The language they used, and everything, made it easier. It made it kind of child friendly I suppose, which is what they should be doing." – P12, male, aged 19, night wetting*

When health professionals failed to do this, participants talked about feeling confused and annoyed.

> *"I always had to ask my mum 'what does that mean? […] he didn't even explain what it was and even my mum didn't really know what it was half the time, because they say things that they think that you'll know, but you don't know. That's frustrating*

For peer review only - http://bmjopen.bmj.com/site/about/guidelines.xhtml

because it's like, I don't know what that means and they're not telling me what that means". – P14, female, age 12, soiling

Due to the sensitive nature of continence problems, appointments often involved questions and discussions about very personal and potentially embarrassing issues. Therefore, building trust and rapport with the clinician was especially important for young people. Continuity of care was highlighted as a factor that helped build rapport and was highly desirable. Participants who reported seeing the same clinician at each appointment described how this helped them to feel more comfortable.

"After my second visit I got to know her a little better and I felt more comfortable every time I went". – P9, male, age 11, day and night wetting

Continuity of care was more likely when participants had been referred to a specialist continence clinic, but was not always guaranteed. More than half of the participants described seeing a different clinician on each visit with no explanation as to why. Participants said that new clinicians were not familiar with their case history, or gave conflicting advice to previous clinicians.

"when we're at the hospital it's always a new person and they always say a different thing to what the other doctor said." – P1, male, age 12, night wetting

Appointments often centred on developing treatment plans to be implemented independently at home by the young person and/or parent. Participants expressed a strong desire to be included in the decision-making process, through being given a rationale for each treatment option and a degree of choice in how to use the treatment plan at home. When health professionals engaged in shared decision-making approaches, participants reported positive appointment experiences.

"I like to know what to do and why I should do it". – P8, male, age 11, night wetting

"He's just really warm and stuff and he just says 'How are you feeling? Do you want to do that? Do you not want to do that?'". – P14, female, age 12, soiling

For peer review only - http://bmjopen.bmj.com/site/about/guidelines.xhtml

Participants described wanting to be treated as an intelligent person and not wanting to be patronised by their clinician. Being seen as an expert in relation to themselves and their body was important.

> *"I like them to treat me as if I'm the person in question, because I'm not stupid, I know I'm not brilliant at medicine or anything, but I can do my research. I know about the medics of it. I know about the treatments that I had, and I know about me, more than anyone". – P2, male, age 14, day and night wetting*

Treatment experiences: the frustration of no magic bullets

Participants often recalled trying a wide range of treatments including urotherapy, dietary changes, medication(s), bedwetting alarms, Botox injections into the bladder, laxatives, suppositories, TENS machine, and hypnotherapy. Almost all participants had tried more than one treatment and had experienced some form of relapse or complete treatment failure. Participants explained that with each failed treatment or relapse, their belief in the success of subsequent treatments diminished.

> *"For the first four months, it was fine…it was a lot better. Then it started to get worse again, and by six or seven months, it was actually worse than it was before I had the [Botox] operation. We went back to do a second one…it was fine for six months. Then it got worse again, and then they called us back to have another one, and we said no. By that point, I was kind of sick of it, and I knew that it wasn't going to work". – P2, male, age 14, day and night wetting*

Belief in treatments seemed to be made much more difficult because of the uncertainty of the cause of young people's continence problems. A minority of participants had been given a medical explanation for their incontinence and reported that this was helpful with respect to the treatments then offered.

> "*I think it made me more comfortable, knowing it was actually a problem and it wasn't just my mum being frantic or something*". – P4, female, age 14, daytime wetting

The majority of participants, however, were given no medical explanation for their incontinence. Participants said that they found this frustrating and confusing.

For peer review only - http://bmjopen.bmj.com/site/about/guidelines.xhtml

"I really struggled, just thinking, 'Why has it become a problem for me?' Because there was not any reason". – P3, female, age 18, daytime wetting

Not being able to match suggested treatments to an underlying cause of the incontinence was also challenging for participants to understand. Other participants had been told there was nothing wrong with them.

"Him saying there's nothing wrong with me, he made me feel quite vulnerable […] it really made me feel bad because we were told that he is the man to see…if he can't do anything about it, like who can?". – P11, male, age 14, daytime wetting

Participants talked extensively about their emotional reactions to treatments saying that the lack of explanation, unpredictability of treatment success combined with experiences of relapse, and treatment failure, led them to feel upset and cynical about the chances of resolution. Many described giving up hope that their problem would ever go away.

"Some of it's obviously a little bit upsetting because I've had so many different medications and trying new things and it hasn't really worked yet, so my hope of it going to getting smaller and smaller". – P16, female, age 15, daytime wetting

For older participants, who had often been undergoing treatment for several years, feelings of pessimism were particularly common.

"For quite a lot of the time the, I felt really really pessimistic about this problem, just like there is absolutely no hope that it's ever going to be better and I'm going to be stuck with this problem forever. Clearly I don't want that to happen, so that really upsets me about it". – P3, female, age 18, daytime wetting

Engagement with treatment: the effects of disappointing results

Participants reported varying levels of engagement with their treatment. Experiencing relapse or treatment failure led many participants to disengage from their treatment, with some admitting that they avoided taking their medication or lied about this to their parents.

"Instead of taking the tablets, I think mum thought I was doing it, say, once a week or something, I was [throwing them away] every day and having problems every day because of that".- P4, female, age 14, daytime wetting

For peer review only - http://bmjopen.bmj.com/site/about/guidelines.xhtml

One participant talked about feeling so frustrated with the lack of improvement in her symptoms that she decided no treatment was going to work and there was no point trying anything new.

> *"I think it was because I'd been on them [the medication] for so long, I didn't feel like they were doing anything. I've never actually tried it [the new medication]. I've never actually properly tried it […] I just decided that it wouldn't work either". – P4, female, age 14, daytime wetting*

Another participant described disengaging from treatment not due to lack of belief in its efficacy, but as an effort to regain her independence.

> *"I think there was a period when I was in year nine [aged 13-14] where I didn't take my medication very well and it just got worse. […] I was just like 'I can do it on my own, I'm an independent woman, I can do what I want". – P15, female, age 14, daytime wetting*

Internalisation and externalisation of the continence problem

Through discussion with participants about their beliefs and attitudes towards their continence problem, two contrasting coping mechanisms were identified; internalisation and externalisation of the continence problem. Participants who internalised their continence problem integrated it into their self-identity. They viewed the problem as a part of their lives and themselves that needed to be actively managed, but not as something that defined or controlled them. Participants who demonstrated internalisation talked about a process of accepting that they had a continence problem, and the need to actively manage it. Taking ownership of their continence problem and embracing it because it made them 'different' and 'unique' were common lines of thought. Others talked about how this attitude helped them to cope with worries about other people finding out about their problem.

> *"I kind of realised that, actually, it's something I can't change, and it's a part of me, so I shouldn't be scared of people knowing. It's not something I've done wrong" – P2, male, age 14, daytime and night wetting*

For peer review only - http://bmjopen.bmj.com/site/about/guidelines.xhtml

In contrast, those who externalised their continence problem saw it as something separate to themselves and their identity, and as an intruder into their lives. Participants who demonstrated externalisation often avoided thinking about their continence problem and talked about distancing themselves from their problem and rejecting it from their self-identity.

> *"…it's just frustrating also because you know what's going to happen. So you kind of always, that's always at the back of your mind. So I do then just try and avoid it [thinking about it] if I can" – P12, male, age 19, night wetting*

In order to deal with stressful social situations, such as sleepovers or school trips, participants said they tried to convince themselves it would just be fine, even if they knew this was highly unlikely.

> *"I think when you have this issue you're trying to put aside, you know the problems but you're trying to get it out of your head, so you will do all you can to convince yourself that you'll be fine even if you won't be". – P11, male, age 14, night wetting*

For peer review only

For peer review only - http://bmjopen.bmj.com/site/about/guidelines.xhtml

DISCUSSION

Appointment experiences

Through interviews with young people with continence problems, we find that positive treatment experiences are characterised by seeing the same clinician at each appointment and by the use of age-appropriate communication. Building a relationship with the same clinician was desirable as it facilitated greater trust and rapport and supported young people to feel more comfortable disclosing highly personal information. Participants expressed a strong desire to be given a full explanation of their continence problem, appropriate to their age and level of understanding. This finding is consistent with studies of clinical experiences of young people with other chronic health problems[33-36]. Young people in this study wanted to be involved in making decisions about their treatment options, rather than the traditional focus on parent-doctor communication in paediatric consultations. Aligned to this, it is now recommended that children and young people should be the primary focus of their own appointments[37, 16]. There is evidence that this facilitates better communication and patient understanding, supports patient autonomy and competence, and promotes successful behaviour change and illness management[38, 39]. Shared decision making between clinician and patient could aid treatment engagement and autonomy in young people and provide them with skills and confidence to transition into adult care.

Factors affecting treatment engagement

The majority of participants in this study had functional incontinence. In the absence of an underlying organic cause of the incontinence, clinicians were often unable to give specific guidance on treatments and prognosis, creating significant illness uncertainty for the participants[40, 41]. This presents challenges since participants often had to try a range of treatments, with varying success[1]. Treatment failures and relapses were common and undermined participants' beliefs in future treatment success and the controllability of their continence problem.

Illness uncertainty and lack belief in the controllability of a health problem are significant barriers to adherence and engagement, because individuals are less likely to follow a

For peer review only - http://bmjopen.bmj.com/site/about/guidelines.xhtml

treatment regime they do not believe will work[42, 43]. Models of health behaviour change consistently highlight the perceived controllability of a health problem as a key factor in predicting health behaviours and treatment adherence[44-45]. Additionally, illness uncertainty has been linked to maladaptive coping, increased psychological distress, depression, and reduced quality of life[46]. When illness uncertainty is an issue, it is crucial that clinicians work to positively engage patients and support them in long-term self-management in order to minimise the impact of their health problem on daily life. Successful self-management also promotes great individual autonomy and can help patients to feel more in control of their bodies[47]. Young people with functional incontinence could also benefit from psychological support and interventions to manage uncertainty and reduce anxiety, such as acceptance commitment therapy[48]. Finding a balance between giving patients hope and being realistic about treatment success is vital in managing patient expectations, and could help to mitigate against negative treatment experiences. By increasing understanding of how treatment failures and relapses affect patients, health professionals can identify potential periods of high-risk non-adherence and take steps to manage this.

Internalisation and externalisation of the problem

Amongst the participants in our sample there was a dichotomy between those who demonstrated internalisation and externalisation of their continence problem. Those who internalised their continence problem had more successfully incorporated this into their self-identity. They recognised it as an ongoing part of their lives, but did not feel restrained or defined by it. In contrast, those who externalised their problem saw it as an external factor that intruded into their life and avoidance coping was common in this group.

Although participants did not directly link these coping strategies to their clinical experiences, the way in which they view their health condition does have significant implications for self-management and adherence to treatment. By viewing continence problems as external to the self, this undermines beliefs in the controllability of the condition, which are linked to treatment adherence[44, 45, 49]. If an individual engages in avoidance coping, they are likely to avoid any stimulus that reminds them of their continence problem, including treatment regimes. Non-adherence increases the risk of treatment failure or relapses, which may further reinforce the belief that the condition is

For peer review only - http://bmjopen.bmj.com/site/about/guidelines.xhtml

uncontrollable, creating a self-perpetuating cycle of disengagement and treatment failure. In contrast, acceptance of a chronic health problem and integration of illness experiences where the condition is viewed as only one characteristic of an individual's life are linked to a greater sense of personal control and proactive management behaviours[50-52].

Whilst within our sample internalisation and externalisation are seen as two different groups, it is likely that within the wider population these responses fall along a continuum. Additionally, individuals may be likely to switch between the two attitudes depending on their experiences. Experiencing relapse or feeling overwhelmed by the condition may promote more externalising attitudes in order to cope. Alternatively, successful treatment and management symptoms may support greater internalisation. Without more exploration of these issues, the exact relationships and mechanism at work are not clear, and further work is needed to better understand this.

LIMITATIONS

Only a small number of participants with soiling were recruited into this study. Young people with soiling problems reported some different experiences to those with urinary incontinence, suggesting that further exploration is needed. Clinicians involved in recruiting to the study reported that young people with soiling were generally less engaged during appointments and would often not admit to having a problem. This led to a problem recruiting these individuals. The participants with soiling either had a medical explanation for their problem (n=1), or were experiencing significant improvements in their symptoms (n=3). Another limitation of the sample was that all the participants in this study were receiving medical help for their continence problem and the majority were seeing specialists. Young people who have not received specialist help for their continence problems are likely to have different experiences to the participants in this study. This study could not explore cultural differences in young people's experiences of continence problems since all but one of the participants identified as white British. In addition, as all participants were from the UK, we do not know if the same issues are experiences in different countries. Socioeconomic background could also impact on experiences of young people with continence problems, but this data was not collected in the current study. As interviews were conducted by Skype and telephone it is possible that some nuances of the

For peer review only - http://bmjopen.bmj.com/site/about/guidelines.xhtml

participants' responses and body language may have been missed. Although this does not change the results, face to face interviews may have yielded an additional level of interpretation and insight.

CONCLUSIONS AND FUTURE DIRECTIONS

Incontinence is challenging to treat and manage, particularly when there is no organic cause. Long-term self-management and helping individuals regain a sense of control is key in minimising the impact of incontinence on daily life. Ensuring positive appointment experiences and engaging young people in their own healthcare is vital. A focus on shared decision-making and ensuring continuity of care are both highly important and facilitate greater engagement with treatment. Clinicians should use age-appropriate language to ensure patient understanding and work to build rapport in order to promote trust and disclosure. Relapses or treatment failure are common and can have a negative emotional impact and undermine beliefs in future treatment success. Assessment of coping styles may a beneficial addition to clinical assessment. For patients who are disengaged or have low treatment adherence, providing psychological support to promote acceptance may be an important first step in successful long-term self-management. These results demonstrate that the same challenges are experienced across the age range and for both organic and non-organic continence problems; this reinforces the importance of addressing these issues.

For peer review only

For peer review only - http://bmjopen.bmj.com/site/about/guidelines.xhtml

REFERENCES

1 Neveus T, von Gontard A, Hoebeke P, et al. The standardisation of terminology of lower urinary tract function in children and adolescents: Report from the standardisation committee of the International Children's Continence Society. *The Journal of Urology,* 2006;176: 314-324

2 Public Health England. Continence Needs Assessment module. 2013. Available: http://atlas.chimat.org.uk/IAS/profiles/needsassessments [Accessed 27th April 2017]

3 NICE. Assumptions used in estimating a population benchmark. 2015. Available: https://www.nice.org.uk/proxy/?sourceurl=http://www.nice.org.uk/usingguidance/commissioningguides/biologicaltherapies/assumptionsusedinestimatingapopulationbenchmark.jsp [Accessed 3 August 2016]

4 NICE. Excellence in continence care. 2015. Available: https://www.england.nhs.uk/commissioning/wp-content/uploads/sites/12/2015/11/EICC-guidance-final-document.pdf [accessed 31 August 2016)

5 Public Health England. Continence needs assessment. 2013. Available: http://atlas.chimat.org.uk/IAS/profiles/profile?profileId=45&geoTypeId= [Accessed 3 August 2016]

6 Joinson C, Grzeda M, von Gontard A, et al. The association between trajectories of bedwetting and daytime wetting in childhood and incontinence and lower urinary tract symptoms in adolescence. *Archives of Disease in Childhood* 2016*;* 101(S1): A187-188

7 Issenman RM, Filmer RB, Gorski PA. A review of bowel and bladder control development in children: how gastrointestinal and urologic conditions relate to problems in toilet training. *Pediatrics* 1999; 103(6 Pt 2): 1346-52.

8 Bower WF. Self-reported effect of childhood incontinence on quality of life. *Journal of Wound Ostomy & Continence Nursing* 2008; 35(6): 617-21.

9 Landgraf JM, Abidari J, Cilento BG, et al. Coping, commitment, and attitude: quantifying the everyday burden of enuresis on children and their families. *Pediatrics* 2004; 113(2): 334-344.

10 von Gontard A, Baeyens D, Van Hoecke E, et al. Psychological and psychiatric issues in urinary and fecal incontinence. *Journal of Urology* 2011; 185(4): 1432-6.

11 Grzeda M, Heron J, von Gontard A, Joinson C. Effects of urinary incontinence on psychosocial outcomes in adolescence. European Child & Adolescent Psychiatry. In press.

12 Filce HG & LaVergne L. Absenteeism, educational plans, and anxiety among children with incontinence and their parents, *Journal of School Health* 2015; 85(4): 241-250

13 Bower WF. Self-reported effect of childhood incontinence on quality of life, *Journal of Wound, Ostomy, and continence nursing* 2008; 36(6): 617-21.

14 von Gontard A. Enuresis. In Rey JM, eds. *IACAPAP e-Textbook of Child and Adolescent Mental Health.* Geneva: International Association for Child and Adolescent Psychiatry and Allied Professions 2012a

15 von Gontard A. Encopresis. In Rey JM, eds. *IACAPAP e-Textbook of Child and Adolescent Mental Health.* Geneva: International Association for Child and Adolescent Psychiatry and Allied Professions 2012b

16 Abrams P, Anderson KE, Brubaker L, et al. Evaluation and treatment of urinary incontinence, pelvic organ prolapse and faecal incontinence, *3rd International Consultation on Incontinence: Recommendations of the International Scientific Committee.* 2005. Available: http://www.ics.org/publications/ici_3/v2.pdf/summary.pdf [accessed 3 August 2016]

17 Buckley BS, Sanders CD, Kwong JS, et al. Conservative treatment for functional daytime urinary incontinence in children. *Cochrane Database of Systematic Reviews* 2016; 9: 1-11.

18 Maternik M, Krzeminska K, Zurowska A. The management of childhood urinary incontinence. *Pediatric Nephrology* 2015; 30(1): 41-50.

For peer review only - http://bmjopen.bmj.com/site/about/guidelines.xhtml

19 Mulders MM, Cobussen-Boekhorst H, de Gier RP, et al. Urotherapy in children: quantitative measurements of daytime urinary incontinence before and after treatment according to the new definitions of the International Children's Continence Society. *Journal of Pediatric Urology* 2011; 7(2): 213-8.

20 Hagstroem S, Rittig S, Kamperis K, et al.. Timer watch assisted urotherapy in children: a randomized controlled trial. *Journal of Urology* 2010; 184(4): 1482-8.

21 NICE. Bedwetting in under 19s, NICE guidelines. 2010. Available at: https://www.nice.org.uk/guidance/cg111/chapter/Key-priorities-for-implementation [accessed 18 August 2016]

22 Burger RE, Mugie SM, Chase J, et al. Management of functional constipation in children with lower urinary tract symptoms: report from the standardization committee of the International Children's Continence Society. *The Journal of Urology* 2013*;* 190: 29-36.

23 NICE. Constipation in children and young people: diagnosis and management, Clinical Guidelines. 2010. Available at: https://www.nice.org.uk/Guidance/CG99 [accessed 19 Sept 2016]

24 Yeung CK, Sreedhar B, Sihoe JD, et al. Differences in characteristics of nighttime enuresis between children and adolescents: a critical appraisal from a large 338 epidemiological study. *British Journal of Urology International* 2006; 97(5): 1069-1073.

25 Swithinbank LV, Brookes ST, Shepherd AM et al. The natural history of urinary symptoms during adolescence. *British Journal of Urology* 1998;81 Suppl 3:90-3.

26 Hellström AL, Hansson E, Hansson S, et al. Micturition habits and incontinence at age 17 - reinvestigation of a cohort studied at age 7. *British Journal of Urology* 1995; 76: 231-234.

27 Kyrklund K, Taskinen S, Rintala RJ, et al. Lower urinary tract symptoms from 353 childhood to adulthood: a population based study of 594 Finnish individuals 4 to 26 354 years old. *Journal of Urology* 2012; 188(2): 588-93.

28 Taddeo D, Egedy M, Frappier JY. Adherence to treatment in adolescents. *Paediatric Child Health* 2008; 13(1): 19-24.

29 Baeyens D, Lierman, A, Roeyes H, Hoebke P, Vande Walle J. Adherence in children with nocturnal enuresis, *Journal of Pediatric Urology* 2008; 5(2): 105-109.

30 Carter B. & Ford K. Researching children's health experiences: The place for participatory, child-centred, arts-based approaches, *Research in Nursing and Health* 2013; 36(1): 95-107.

31 Braun V. & Clarke V. Using thematic analysis in psychology. *Qualitative Research in* Psychology 2006; 3(2): 77-101.

32 Tracey SJ. Qualitative quality: Eight "Big-Tent" criteria for excellent qualitative research, *Qualitative Inquiry* 2010; 16(10): 837-851.

33 de Winter M, Baerveldt C, Kooistra J. Enabling children: Participation as a new perspective on child-health promotion. *Child Care Health Development* 2002; 28:109–16.

34 Holtzheimer L, Mohay H, Masters IB. Educating young children about asthma: Comparing the effectiveness of developmentally appropriate asthma education videotape and picture book. *Child Care Health Development* 1998; 24: 85–99.

35 Ginsburg KR, Menapace AS, Slap GB. Factors affecting the decision to seek health care: The voice of adolescents. *Pediatrics* 1997; 100: 922–30.

36 Freed LH, Ellen JM, Irwin CE, et al. Determinants of adolescent satisfaction with health care providers and intentions to keep follow-up appointments. *Journal of Adolescent* Health 1998; 22: 475–9.

37 Department for Health. Getting the right start: The national service framework for children, young people and maternity services – Standards for hospital services. London: Stationary Office 2008.

For peer review only - http://bmjopen.bmj.com/site/about/guidelines.xhtml

38 Deci EL & Ryan RM. Intrinsic Motivation and Self-Determination in Human Behaviour. New York: Plenum Publishing Co. 1985.

39 Deci EL & Ryan RM. Self-determination theory: A macrotheory of human motivation, development, and health. *Psychologie canadienne* 2008; 49(3): 182-185.

40 Mishel MH. Uncertainty in illness. *Image: The Journal of Nursing Scholarship* 1988; 20(4): 225–232.

41 Mishel MH. Reconceptualization of the uncertainty in illness theory. Image: *Journal of Nursing Scholarship* 1990; 22(4): 256-262.

42 Ballif M, Ledergerber B, Battegay, M. Impact of previous virological treatment failures and adherence on the outcome of antitretroviral therapy in 2007, *PLoS ONE 2009;* 4(12): e8275

43 Mann D, Ponieman D, Leventhal H, et al. Predictors of adherence to diabetes medications: The role of disease and medication beliefs. *Journal of Behavioural Medicine* 2009; 32(3): 278-284.

44 Zugelj U, Zupancic M, Komidar L, et al. Self-reported adherence beahviours in adolescent hypertensive patients: the role of illness representations and personality. *Journal of Paediatric Psychology* 2010: 1-12.

45 Leventhal H, Brissette I & Leventhal EA. The common-sense model of self-regulation of health and illness. In: Cameron LD, Leventhal H, eds. The Self-Regulation of Health and Illness behaviour. London: Routledge 2003: 42-65.

46 Wright LJ, Afari N. & Zautra, A. The illness uncertainty concept: a review. *Current Pain and Headache Reports* 2009; 13: 133-138.

47 Williams GC, McGregor HA, Zeldman A, et al. Testing a self-determination theory process model for promoting glycemic control through diabetes self-management. *Health Psychology* 2004; 23(1): 58-66.

48 Hayes SC, Luoma JB, Bond FW, et al. Acceptance and commitment therapy: model, processes and outcomes. *Behaviour Research and Therapy* 2006; 44: 1-25.

49 Leventhal H, Diefenbach M. & Leventhal E. Illness cognition: Using common sense to understand treatment adherence and affect cognition interactions. *Cognitive Therapy and Research* 1992; 16: 143-163.

50 Li L. & Moore D. Acceptance of disability and its correlates. *Journal of Social Psychology* 1998; 138: 13-25.

51 Stuifbergen A, Becker H, Blozis, S, et al. Conceptualization of development of the acceptance of chronic health conditions scale. *Issues in Mental Health Nursing* 2008; 29: 101-114.

52 Karademas EC, Tsagaraki A. & Lambrou N. Illness acceptance, hospitalization stress, and subjective health in a sample of chronic patients admitted to hospital. *Journal of Health Psychology 2009;* 14:1243–1250

For peer review only

COREQ (COnsolidated criteria for REporting Qualitative research) Checklist

A checklist of items that should be included in reports of qualitative research. You must report the page number in your manuscript where you consider each of the items listed in this checklist. If you have not included this information, either revise your manuscript accordingly before submitting or note N/A.

Topic	Item No.	Guide Questions/Description	Reported on Page No.
**Domain 1: Research team and reflexivity**			
Personal characteristics			
Interviewer/facilitator	1	Which author/s conducted the interview or focus group?	8
Credentials	2	What were the researcher's credentials? E.g. PhD, MD	8
Occupation	3	What was their occupation at the time of the study?	8
Gender	4	Was the researcher male or female?	8
Experience and training	5	What experience or training did the researcher have?	8
Relationship with participants			
Relationship established	6	Was a relationship established prior to study commencement?	n/a
Participant knowledge of the interviewer	7	What did the participants know about the researcher? e.g. personal goals, reasons for doing the research	n/a
Interviewer characteristics	8	What characteristics were reported about the inter viewer/facilitator? e.g. Bias, assumptions, reasons and interests in the research topic	n/a
**Domain 2: Study design**			
Theoretical framework			
Methodological orientation and Theory	9	What methodological orientation was stated to underpin the study? e.g. grounded theory, discourse analysis, ethnography, phenomenology, content analysis	8
Participant selection			
Sampling	10	How were participants selected? e.g. purposive, convenience, consecutive, snowball	7
Method of approach	11	How were participants approached? e.g. face-to-face, telephone, mail, email	7
Sample size	12	How many participants were in the study?	7
Non-participation	13	How many people refused to participate or dropped out? Reasons?	7
Setting			
Setting of data collection	14	Where was the data collected? e.g. home, clinic, workplace	7
Presence of non-participants	15	Was anyone else present besides the participants and researchers?	n/a
Description of sample	16	What are the important characteristics of the sample? e.g. demographic data, date	9
Data collection			
Interview guide	17	Were questions, prompts, guides provided by the authors? Was it pilot tested?	7-8
Repeat interviews	18	Were repeat inter views carried out? If yes, how many?	n/a
Audio/visual recording	19	Did the research use audio or visual recording to collect the data?	8
Field notes	20	Were field notes made during and/or after the inter view or focus group?	n/a
Duration	21	What was the duration of the inter views or focus group?	8
Data saturation	22	Was data saturation discussed?	n/a
Transcripts returned	23	Were transcripts returned to participants for comment and/or	n/a

For peer review only

Topic	Item No.	Guide Questions/Description	Reported on Page No.
		correction?	
**Domain 3: analysis and findings**			
Data analysis			
Number of data coders	24	How many data coders coded the data?	8
Description of the coding tree	25	Did authors provide a description of the coding tree?	n/a
Derivation of themes	26	Were themes identified in advance or derived from the data?	10
Software	27	What software, if applicable, was used to manage the data?	8
Participant checking	28	Did participants provide feedback on the findings?	n/a
Reporting			
Quotations presented	29	Were participant quotations presented to illustrate the themes/findings? Was each quotation identified? e.g. participant number	10-15
Data and findings consistent	30	Was there consistency between the data presented and the findings?	10-15
Clarity of major themes	31	Were major themes clearly presented in the findings?	10-15
Clarity of minor themes	32	Is there a description of diverse cases or discussion of minor themes?	10-15

Developed from: Tong A, Sainsbury P, Craig J. Consolidated criteria for reporting qualitative research (COREQ): a 32-item checklist for interviews and focus groups. *International Journal for Quality in Health Care*. 2007. Volume 19, Number 6: pp. 349 – 357

Once you have completed this checklist, please save a copy and upload it as part of your submission. DO NOT include this checklist as part of the main manuscript document. It must be uploaded as a separate file.

For peer review only

BMJ Open

‘What does that mean?’: A qualitative exploration of the primary and secondary clinical care experiences of young people with continence problems in the UK

Journal:	*BMJ Open*
Manuscript ID	bmjopen-2016-015544.R2
Article Type:	Research
Date Submitted by the Author:	07-Jun-2017
Complete List of Authors:	Whale, Katie; University of Bristol, Centre for Child and Adolescent Health<br>Cramer, Helen; Centre for Academic Primary Care, School of Social and Community Medicine<br>Wright, Anne; Evelina London Children's Hospital<br>Sanders, Caroline ; University of Northern British Columbia<br>Joinson, Carol; University of Bristol, Centre for Child and Adolescent Health; School of Social and Community Medicine, University of Bristol
**Primary Subject Heading**:	Urology
Secondary Subject Heading:	Paediatrics, Qualitative research
Keywords:	Urinary incontinences < UROLOGY, Fecal incontinence, PAEDIATRICS

SCHOLARONE™ Manuscripts

For peer review only

'What does that mean?': A qualitative exploration of the primary and secondary clinical care experiences of young people with continence problems in the UK

Whale K[1], Cramer H[2], Wright, A[3], Sanders, C[4] & Joinson C[1]

[1] Centre for Child and Adolescent Health, School of Social and Community Medicine, University of Bristol

[2] Centre for Academic Primary Care, School of Social and Community Medicine, University of Bristol

[3] Evelina London Children's Hospital, Guy's and St Thomas' NHS Foundation Trust, London

[4] University of Northern British Columbia, Canada

Corresponding author

Dr Katie Whale

Centre for Child and Adolescent Health

School of Social and Community Medicine

University of Bristol

Oakfield House

Oakfield Grove

Bristol

BS8 2BN

Email: Katie.whale@bristol.ac.uk

Phone: 0117 3313328

Data sharing statement

Data from the study is classified as controlled access. No participant consent was given to share their interview data, therefore no further data from this study is available.

For peer review only - http://bmjopen.bmj.com/site/about/guidelines.xhtml

Abstract

Objectives: To explore the clinical care experiences of young people with continence problems.

Design: In-depth semi-structured qualitative interviews were conducted by Skype and telephone, with the addition of art-based participatory research techniques. Transcripts were analysed using inductive thematic analysis.

Setting: Primary and secondary care in the UK.

Participants: We interviewed 20 participants (nine females, 11 males) aged 11-20. There were six participants with bedwetting alone, five with daytime wetting alone, five with combined (day and night) wetting, and four with soiling.

Results: We identified four themes: appointment experiences, treatment experiences, engagement with treatment, and internalisation and externalisation of the continence problem. Patient-focused appointments using age-appropriate language were highly desirable. Continuity of care was highlighted as an important aspect of positive clinical experiences, however this was found to be rare with many participants seeing a different person on each visit. Participants had tried a wide range of treatments for their continence problems with varying degrees of success. Relapse and treatment failure were common. Experiencing relapse was distressing and diminished participants' belief in the success of future treatments, and undermined adherence. Participants would be seen to adopt two opposing coping strategies for dealing with their continence problem, internalisation and externalisation.

Conclusion: Incontinence in young people is challenging to manage. Young people may need to try a range of treatments before their symptoms improve. Due to challenges in treatment there is an increased risk of poor adherence. During patient-focused appointments clinicians should work to build rapport with patients and use age-appropriate language. Involving young people in their own care decisions is important. The way in which young people understand their continence problem can influence their coping strategies and adherence to treatment regimes.

For peer review only - http://bmjopen.bmj.com/site/about/guidelines.xhtml

Strengths and limitations of the study

- In-depth qualitative interviews provide a unique insight into the experiences of young people with incontinence
- Due to the sensitive nature of this topic, interviews were combined with an arts-based participatory approach to facilitate greater disclosure and gain a better insight into young people's lives
- Skype and telephone interviews allowed access to participants from a wide geographical area
- Telephone interviews presented challenges in building rapport and body language with the participants, which may have hindered some personal disclosure

This research was funded by a grant from the Medical Research Council (Increasing understanding of risk factors and outcomes associated with continence problems in children and adolescents. MRC reference: MR/L007231/1).

We have read and understood BMJ policy on declaration of interests and declare that we have no competing interests

Author contributions

Katie Whale: Lead researcher, design of qualitative work, conducting interviews, analysis of interview data, writing and preparing manuscript

Helen Cramer: Design of qualitative work, analysis of a sub-set of interviews and input into analysis process, member of the project steering group, revising manuscript critically for important intellectual content

Anne Wright: Recruitment of participants, contribution to interview design, contribution to interpretation of themes, member of the project steering group, revising manuscript critically for important intellectual content

For peer review only - http://bmjopen.bmj.com/site/about/guidelines.xhtml

Caroline Sanders: Contribution to interpretation of themes, revising manuscript critically for important intellectual content

Carol Joinson: Principal investigator, obtaining ethical approval for the study, design of qualitative work, analysis of a sub-set of interviews and input into analysis process, revising manuscript critically for important intellectual content

For peer review only

For peer review only - http://bmjopen.bmj.com/site/about/guidelines.xhtml

INTRODUCTION

Continence problems (daytime wetting, bedwetting, and soiling) [1] are among the most common chronic conditions of childhood. In the UK it is estimated that 900,000 children and young people experience some form of continence problem[2]. This is, however, likely to be an underestimate as the stigma associated with incontinence often prevents individuals from seeking treatment[3, 4]. Epidemiological studies estimate the prevalence of incontinence in 11–20 year olds to be around 2–3% for daytime wetting, 2–2.5% for bedwetting and 1–1.5% for soiling[5, 6]. Incontinence in children and young people has been linked to high rates of emotional distress, poor self-image, relationship problems and unhappiness at school[7-12]. Poor management of continence problems has an adverse impact on quality of life and self-esteem during childhood and adolescence[13].

A minority of children and young people have an organic (neurological, structural or anatomic) cause for their incontinence, but the vast majority of paediatric continence problems are functional (no underlying organic cause)[14, 15, 1]. Uncertainty surrounding individual responses to treatments for functional incontinence and varying levels of access to paediatric continence services are major factors affecting patients with incontinence[2]. Treatment for functional incontinence is varied and there is often no singularly effective treatment. As a consequence, patients may need to try a series of different approaches to manage or improve their incontinence symptoms[16, 17]. The first-line treatment for daytime wetting is often urotherapy, involving a structured regime of toileting and fluid intake, sometimes combined with medication[18-20]. Initial treatments for bedwetting include the bedwetting alarm and/or medication (e.g. desmopressin)[16, 21, 14]. Constipation is a common cause of soiling and can exacerbate urinary incontinence due to pressure on the bladder. The first stage of treatment for soiling usually involves laxatives or stool softeners in addition to a toileting programme and, in some cases, suppositories[22, 15, 23].

Although many children experience a natural resolution of their incontinence with age, there is evidence that those with severe incontinence are at risk of their problems persisting into adolescence [6, 24-27]. Often when curative treatment fails there is a refocusing on symptom management as the child continues to mature and enters into adolescence. Adolescence can be a particularly challenging time for the management of chronic health

For peer review only - http://bmjopen.bmj.com/site/about/guidelines.xhtml

problems and is frequently associated with lower levels of treatment adherence[28]. Due to the challenges of treatment, young people with persistent continence problems may see minimal improvements and may need to follow treatment regimes for extended periods before seeing any changes. Previous work has shown that adolescents often struggle to adhere to treatments that show few immediate benefits[28,29].

Little research has been published on the primary and secondary care experiences of young people with continence problems, especially with regard to their experiences of treatment and management of their continence problems. The overall aim of the project was to explore the impact of continence problems on young people and the specific focus of this paper is on their experiences of primary and secondary care.

For peer review only

For peer review only - http://bmjopen.bmj.com/site/about/guidelines.xhtml

METHODS

Participants were recruited through five secondary care paediatric continence clinics (four in England and one in Scotland), and through an advertisement on a paediatric continence charity website (The Children's Bowel and Bladder Charity, www.eric.org.uk).
Eligible participants were between 11 and 20 years, currently experiencing continence problems (bedwetting, daytime wetting, or soiling) or who previously experienced these problems after the age of 10, and able to speak and understand English.

Participants attending paediatric continence clinics were given a study information pack by their clinician. Those recruited through the advertisement were sent an information pack by the research team. Ethical approval was given by the National Research Ethics Service Committee South West - Central Bristol (14/SW/0059). The researcher contacted all interested participants by phone to describe the study, the motivation for the work, and answer any questions. All participants were unknown to the researcher prior to the commencement of the study. In total 45 potential participants were identified (36 through clinics and 9 through ERIC). 25 participants were not interviewed for the following reasons: 12 unable to contact, 5 not interested in taking part, 8 withdrawn.

In-depth semi-structured interviews were conducted with 20 young people between February 2015 and January 2016 (see table 1 for participant characteristics). Recruitment and final sample size were guided by concept of 'information power'[30]. Given the specificity of the sample, broad study aim, and strong interview dialogue, a sample size of 20 participants was considered appropriate.

Participants were given the option to be interviewed by telephone, Skype, or face to face (for participants within a 40 mile radius of Bristol). Among the participants, 11 were interviewed by Skype and nine by telephone. No participants were interviewed face to face (those given this option preferred to be interviewed by Skype or telephone). No repeat interviews were conducted. Four participants chose to have their parent/guardian present during the interview, seven were in a communal area of their house, and nine chose to conduct the interview alone in a private room.

For peer review only - http://bmjopen.bmj.com/site/about/guidelines.xhtml

A topic guide was developed based on previous literature on the treatment, management and impact of continence problems in children and young people. The topic guide covered issues such as attending appointments, treatment experiences, school or work, and thoughts and feelings about their continence problem. Due to the exploratory nature of the study the interviews were semi-structured. The topic guide was used as a starting point for discussion, with flexibility to discuss novel areas introduced by the participants.

An arts-based participatory approach was used in the interviews. This approach is considered appropriate for children and young people since it provides additional narratives through which personal experiences can be explored[31]. A participant activity pack was developed for use prior to and during the interviews containing a graphic representation of each possible topic area, allowing the participants to write or draw their thoughts in advance, if they wished to. Participants were sent the pack in advance of their interview and were given a verbal explanation of how it could be used during the initial phone call.

All participants provided written informed consent, for ages 16 and above, or parental written consent and child assent for those below 16 years. Interviews were conducted by one female researcher with extensive experience of qualitative work (KW: DHealthPsy, Senior Research Associate) and lasted between 34 and 99 minutes (mean 65 minutes). The researcher made detailed field notes after each interview, including details about the environment, participant demeanour, and personal reflections. Data were recorded using an encrypted audio recorder. Data collection and analysis were conducted in parallel after completion of the first five interviews. Early analysis was used to refine the topic guide and to further explore emerging areas of interest. Interviews were audio recorded, fully transcribed, and imported into the software package NVivo10. Inductive thematic analysis was carried out following guidelines of Braun and Clarke[32]. Following completion of the first five interviews, each transcript was read and the data were free-coded across all transcripts. A selection of three transcripts were also independently free coded by the study team (CJ and HC). Codes were discussed and compared with all members of the team in order to further refine coding and to maximise rigor[33]. An initial set of agreed codes were set up within the NVivo10 database and any new codes identified from further interviews were discussed within the team and added to the coding framework. At the end of the analysis

For peer review only - http://bmjopen.bmj.com/site/about/guidelines.xhtml

process participants were sent a summary of the findings and were given the opportunity to provide feedback and comments. A strong theme was identified from the data on young people's experiences of clinical care.

Sample description

In total, 20 interviews were carried out with children and young people aged 11-20 years. 17 were recruited through paediatric continence clinics and three through the ERIC advertisement. Three had organic incontinence and 17 had non-organic incontinence. Table 1 provides a full overview of participant characteristics.

Table 1: Participant characteristics

**Participant ID no.**	**Gender**		**Continence problem**	**Method of interview**
1	M		Night wetting	Skype
2	M		Day and night wetting	Telephone
3	F		Daytime wetting	Skype
4	F		Daytime wetting	Skype
5	F		Day and night wetting	Skype
6	M		Day and night wetting	Skype
7	M		Night wetting	Telephone
8	M		Night wetting	Telephone
9	M		Day and night wetting	Telephone
10	F		Night wetting	Skype
11	M		Daytime wetting	Skype
12	M		Night wetting	Telephone
13	F		Soiling	Skype
14	F		Soiling	Skype
15	F		Daytime wetting	Skype
16	F		Daytime wetting	Telephone
17	M		Soiling	Telephone
18	F		Night wetting	Telephone
19	M		Soiling	Telephone
20	M		Day and night wetting	Skype

For peer review only - http://bmjopen.bmj.com/site/about/guidelines.xhtml

RESULTS

Themes

Three themes directly linking to clinical care experiences were identified from the data: *appointment experiences*, *treatment experiences*, and *engagement with treatment*. A fourth theme was identified from data that was not directly linked to clinical experiences but was felt to have important implications for self-management and adherence; *internalisation and externalisation of the continence problem*.

For peer review only

Appointment experiences: what makes a good appointment?

Participants in the study had seen a range of health professionals during their treatment journey including general practitioners (GPs), community and school nurses, paediatricians, urologists, and paediatric continence specialists. Participants' first contact with health services was most commonly with a GP or a community or school nurse. Participants recruited through the paediatric continence clinics had the experience of attending a specialist service, whilst those recruited through the online advertisement had primarily seen their GP, or a small number had seen a general paediatrician.

For all participants, the most important factor that influenced their appointment experiences was the communication style used by the clinician, specifically, whether the clinician used patient-centred communication and age-appropriate language. Participants expressed a strong desire for the clinician to include them in the conversation and talk to them directly (rather than to their parent), and to use language that they could understand.

> *"The language they used, and everything, made it easier. It made it kind of child friendly I suppose, which is what they should be doing." – P12, male, aged 19, night wetting*

When health professionals failed to do this, participants talked about feeling confused and annoyed.

> *"I always had to ask my mum 'what does that mean? […] he didn't even explain what it was and even my mum didn't really know what it was half the time, because they say things that they think that you'll know, but you don't know. That's frustrating*

For peer review only - http://bmjopen.bmj.com/site/about/guidelines.xhtml

because it's like, I don't know what that means and they're not telling me what that means". – P14, female, age 12, soiling

Due to the sensitive nature of continence problems, appointments often involved questions and discussions about very personal and potentially embarrassing issues. Therefore, building trust and rapport with the clinician was especially important for young people. Continuity of care was highlighted as a factor that helped build rapport and was highly desirable. Participants who reported seeing the same clinician at each appointment described how this helped them to feel more comfortable.

"After my second visit I got to know her a little better and I felt more comfortable every time I went". – P9, male, age 11, day and night wetting

Continuity of care was more likely when participants had been referred to a specialist continence clinic, but was not always guaranteed. More than half of the participants described seeing a different clinician on each visit with no explanation as to why. Participants said that new clinicians were not familiar with their case history, or gave conflicting advice to previous clinicians.

"when we're at the hospital it's always a new person and they always say a different thing to what the other doctor said." – P1, male, age 12, night wetting

Appointments often centred on developing treatment plans to be implemented independently at home by the young person and/or parent. Participants expressed a strong desire to be included in the decision-making process, through being given a rationale for each treatment option and a degree of choice in how to use the treatment plan at home. When health professionals engaged in shared decision-making approaches, participants reported positive appointment experiences.

"I like to know what to do and why I should do it". – P8, male, age 11, night wetting

"He's just really warm and stuff and he just says 'How are you feeling? Do you want to do that? Do you not want to do that?'". – P14, female, age 12, soiling

For peer review only - http://bmjopen.bmj.com/site/about/guidelines.xhtml

Participants described wanting to be treated as an intelligent person and not wanting to be patronised by their clinician. Being seen as an expert in relation to themselves and their body was important.

> *"I like them to treat me as if I'm the person in question, because I'm not stupid, I know I'm not brilliant at medicine or anything, but I can do my research. I know about the medics of it. I know about the treatments that I had, and I know about me, more than anyone". – P2, male, age 14, day and night wetting*

Treatment experiences: the frustration of no magic bullets

Participants often recalled trying a wide range of treatments including urotherapy, dietary changes, medication(s), bedwetting alarms, Botox injections into the bladder, laxatives, suppositories, TENS machine, and hypnotherapy. Almost all participants had tried more than one treatment and had experienced some form of relapse or complete treatment failure. Participants explained that with each failed treatment or relapse, their belief in the success of subsequent treatments diminished.

> *"For the first four months, it was fine…it was a lot better. Then it started to get worse again, and by six or seven months, it was actually worse than it was before I had the [Botox] operation. We went back to do a second one…it was fine for six months. Then it got worse again, and then they called us back to have another one, and we said no. By that point, I was kind of sick of it, and I knew that it wasn't going to work". – P2, male, age 14, day and night wetting*

Belief in treatments seemed to be made much more difficult because of the uncertainty of the cause of young people's continence problems. A minority of participants had been given a medical explanation for their incontinence and reported that this was helpful with respect to the treatments then offered.

> *"I think it made me more comfortable, knowing it was actually a problem and it wasn't just my mum being frantic or something*". – P4, female, age 14, daytime wetting

The majority of participants, however, were given no medical explanation for their incontinence. Participants said that they found this frustrating and confusing.

For peer review only - http://bmjopen.bmj.com/site/about/guidelines.xhtml

> *"I really struggled, just thinking, 'Why has it become a problem for me?' Because there was not any reason*". – P3, female, age 18, daytime wetting

Not being able to match suggested treatments to an underlying cause of the incontinence was also challenging for participants to understand. Other participants had been told there was nothing wrong with them.

> *"Him saying there's nothing wrong with me, he made me feel quite vulnerable […] it really made me feel bad because we were told that he is the man to see…if he can't do anything about it, like who can?"*. – P11, male, age 14, daytime wetting

Participants talked extensively about their emotional reactions to treatments saying that the lack of explanation, unpredictability of treatment success combined with experiences of relapse, and treatment failure, led them to feel upset and cynical about the chances of resolution. Many described giving up hope that their problem would ever go away.

> *"Some of it's obviously a little bit upsetting because I've had so many different medications and trying new things and it hasn't really worked yet, so my hope of it going to getting smaller and smaller". – P16, female, age 15, daytime wetting*

For older participants, who had often been undergoing treatment for several years, feelings of pessimism were particularly common.

> *"For quite a lot of the time the, I felt really really pessimistic about this problem, just like there is absolutely no hope that it's ever going to be better and I'm going to be stuck with this problem forever. Clearly I don't want that to happen, so that really upsets me about it". – P3, female, age 18, daytime wetting*

Engagement with treatment: the effects of disappointing results

Participants reported varying levels of engagement with their treatment. Experiencing relapse or treatment failure led many participants to disengage from their treatment, with some admitting that they avoided taking their medication or lied about this to their parents.

> *"Instead of taking the tablets, I think mum thought I was doing it, say, once a week or something, I was [throwing them away] every day and having problems every day because of that".- P4, female, age 14, daytime wetting*

For peer review only - http://bmjopen.bmj.com/site/about/guidelines.xhtml

One participant talked about feeling so frustrated with the lack of improvement in her symptoms that she decided no treatment was going to work and there was no point trying anything new.

> *"I think it was because I'd been on them [the medication] for so long, I didn't feel like they were doing anything. I've never actually tried it [the new medication]. I've never actually properly tried it […] I just decided that it wouldn't work either". – P4, female, age 14, daytime wetting*

Another participant described disengaging from treatment not due to lack of belief in its efficacy, but as an effort to regain her independence.

> *"I think there was a period when I was in year nine [aged 13-14] where I didn't take my medication very well and it just got worse. […] I was just like 'I can do it on my own, I'm an independent woman, I can do what I want". – P15, female, age 14, daytime wetting*

Internalisation and externalisation of the continence problem

Through discussion with participants about their beliefs and attitudes towards their continence problem, two contrasting coping mechanisms were identified; internalisation and externalisation of the continence problem. Participants who internalised their continence problem integrated it into their self-identity. They viewed the problem as a part of their lives and themselves that needed to be actively managed, but not as something that defined or controlled them. Participants who demonstrated internalisation talked about a process of accepting that they had a continence problem, and the need to actively manage it. Taking ownership of their continence problem and embracing it because it made them 'different' and 'unique' were common lines of thought. Others talked about how this attitude helped them to cope with worries about other people finding out about their problem.

> *"I kind of realised that, actually, it's something I can't change, and it's a part of me, so I shouldn't be scared of people knowing. It's not something I've done wrong" – P2, male, age 14, daytime and night wetting*

For peer review only - http://bmjopen.bmj.com/site/about/guidelines.xhtml

For peer review only

In contrast, those who externalised their continence problem saw it as something separate to themselves and their identity, and as an intruder into their lives. Participants who demonstrated externalisation often avoided thinking about their continence problem and talked about distancing themselves from their problem and rejecting it from their self-identity.

> *"…it's just frustrating also because you know what's going to happen. So you kind of always, that's always at the back of your mind. So I do then just try and avoid it [thinking about it] if I can" – P12, male, age 19, night wetting*

In order to deal with stressful social situations, such as sleepovers or school trips, participants said they tried to convince themselves it would just be fine, even if they knew this was highly unlikely.

> *"I think when you have this issue you're trying to put aside, you know the problems but you're trying to get it out of your head, so you will do all you can to convince yourself that you'll be fine even if you won't be". – P11, male, age 14, night wetting*

For peer review only - http://bmjopen.bmj.com/site/about/guidelines.xhtml

DISCUSSION

Appointment experiences

Through interviews with young people with continence problems, we find that positive treatment experiences are characterised by seeing the same clinician at each appointment and by the use of age-appropriate communication. Building a relationship with the same clinician was desirable as it facilitated greater trust and rapport and supported young people to feel more comfortable disclosing highly personal information. Participants expressed a strong desire to be given a full explanation of their continence problem, appropriate to their age and level of understanding. This finding is consistent with studies of clinical experiences of young people with other chronic health problems[34-37]. Young people in this study wanted to be involved in making decisions about their treatment options, rather than the traditional focus on parent-doctor communication in paediatric consultations. Aligned to this, it is now recommended that children and young people should be the primary focus of their own appointments[38, 16]. There is evidence that this facilitates better communication and patient understanding, supports patient autonomy and competence, and promotes successful behaviour change and illness management[39, 40]. Shared decision making between clinician and patient could aid treatment engagement and autonomy in young people and provide them with skills and confidence to transition into adult care.

Factors affecting treatment engagement

The majority of participants in this study had functional incontinence (n= 17). In the absence of an underlying organic cause of the incontinence, clinicians were often unable to give specific guidance on treatments and prognosis, creating significant illness uncertainty for the participants[41, 42]. This presents challenges since participants often had to try a range of treatments, with varying success[1]. Treatment failures and relapses were common and undermined participants' beliefs in future treatment success and the controllability of their continence problem.

Illness uncertainty and lack belief in the controllability of a health problem are significant barriers to adherence and engagement, because individuals are less likely to follow a

For peer review only - http://bmjopen.bmj.com/site/about/guidelines.xhtml

treatment regime they do not believe will work[43, 44]. Models of health behaviour change consistently highlight the perceived controllability of a health problem as a key factor in predicting health behaviours and treatment adherence[45-46]. Additionally, illness uncertainty has been linked to maladaptive coping, increased psychological distress, depression, and reduced quality of life[47]. When illness uncertainty is an issue, it is crucial that clinicians work to positively engage patients and support them in long-term self-management in order to minimise the impact of their health problem on daily life. Successful self-management also promotes great individual autonomy and can help patients to feel more in control of their bodies[48]. Young people with functional incontinence could also benefit from psychological support and interventions to manage uncertainty and reduce anxiety, such as acceptance commitment therapy[49]. Finding a balance between giving patients hope and being realistic about treatment success is vital in managing patient expectations, and could help to mitigate against negative treatment experiences. By increasing understanding of how treatment failures and relapses affect patients, health professionals can identify potential periods of high-risk non-adherence and take steps to manage this.

Internalisation and externalisation of the problem

Amongst the participants in our sample there was a dichotomy between those who demonstrated internalisation and externalisation of their continence problem. Those who internalised their continence problem had more successfully incorporated this into their self-identity. They recognised it as an ongoing part of their lives, but did not feel restrained or defined by it. In contrast, those who externalised their problem saw it as an external factor that intruded into their life and avoidance coping was common in this group.

Although participants did not directly link these coping strategies to their clinical experiences, the way in which they view their health condition does have significant implications for self-management and adherence to treatment. By viewing continence problems as external to the self, this undermines beliefs in the controllability of the condition, which are linked to treatment adherence[45, 46, 50]. If an individual engages in avoidance coping, they are likely to avoid any stimulus that reminds them of their continence problem, including treatment regimes. Non-adherence increases the risk of treatment failure or relapses, which may further reinforce the belief that the condition is

For peer review only - http://bmjopen.bmj.com/site/about/guidelines.xhtml

For peer review only

uncontrollable, creating a self-perpetuating cycle of disengagement and treatment failure. In contrast, acceptance of a chronic health problem and integration of illness experiences where the condition is viewed as only one characteristic of an individual's life are linked to a greater sense of personal control and proactive management behaviours[51-53].

Whilst within our sample internalisation and externalisation are seen as two different groups, it is likely that within the wider population these responses fall along a continuum. Additionally, individuals may be likely to switch between the two attitudes depending on their experiences. Experiencing relapse or feeling overwhelmed by the condition may promote more externalising attitudes in order to cope. Alternatively, successful treatment and management symptoms may support greater internalisation. Without more exploration of these issues, the exact relationships and mechanism at work are not clear, and further work is needed to better understand this.

LIMITATIONS

Only a small number of participants with soiling were recruited into this study. Young people with soiling problems reported some different experiences to those with urinary incontinence, suggesting that further exploration is needed. Clinicians involved in recruiting to the study reported that young people with soiling were generally less engaged during appointments and would often not admit to having a problem. This led to a problem recruiting these individuals. The participants with soiling either had a medical explanation for their problem (n=1), or were experiencing significant improvements in their symptoms (n=3). Another limitation of the sample was that all the participants in this study were receiving medical help for their continence problem and the majority were seeing specialists. Young people who have not received specialist help for their continence problems are likely to have different experiences to the participants in this study. This study could not explore cultural differences in young people's experiences of continence problems since all but one of the participants identified as white British. In addition, as all participants were from the UK, we do not know if the same issues are experiences in different countries. Socioeconomic background could also impact on experiences of young people with continence problems, but this data was not collected in the current study. As interviews were conducted by Skype and telephone it is possible that some nuances of the

For peer review only - http://bmjopen.bmj.com/site/about/guidelines.xhtml

participants' responses and body language may have been missed. Although this does not change the results, face to face interviews may have yielded an additional level of interpretation and insight.

CONCLUSIONS AND FUTURE DIRECTIONS

Incontinence is challenging to treat and manage, particularly when there is no organic cause. Long-term self-management and helping individuals regain a sense of control is key in minimising the impact of incontinence on daily life. Ensuring positive appointment experiences and engaging young people in their own healthcare is vital. A focus on shared decision-making and ensuring continuity of care are both highly important and facilitate greater engagement with treatment. Clinicians should use age-appropriate language to ensure patient understanding and work to build rapport in order to promote trust and disclosure. Relapses or treatment failure are common and can have a negative emotional impact and undermine beliefs in future treatment success. Assessment of coping styles may a beneficial addition to clinical assessment. For patients who are disengaged or have low treatment adherence, providing psychological support to promote acceptance may be an important first step in successful long-term self-management. These results demonstrate that the same challenges are experienced across the age range and for both organic and non-organic continence problems; this reinforces the importance of addressing these issues.

For peer review only

For peer review only - http://bmjopen.bmj.com/site/about/guidelines.xhtml

REFERENCES

1 Neveus T, von Gontard A, Hoebeke P, et al. The standardisation of terminology of lower urinary tract function in children and adolescents: Report from the standardisation committee of the International Children's Continence Society. *The Journal of Urology,* 2006;176: 314-324

2 Public Health England. Continence Needs Assessment module. 2013. Available: http://atlas.chimat.org.uk/IAS/profiles/needsassessments [Accessed 27th April 2017]

3 NICE. Assumptions used in estimating a population benchmark. 2015. Available: https://www.nice.org.uk/proxy/?sourceurl=http://www.nice.org.uk/usingguidance/commissioningguides/biologicaltherapies/assumptionsusedinestimatingapopulationbenchmark.jsp [Accessed 3 August 2016]

4 NICE. Excellence in continence care. 2015. Available: https://www.england.nhs.uk/commissioning/wp-content/uploads/sites/12/2015/11/EICC-guidance-final-document.pdf [accessed 31 August 2016)

5 Public Health England. Continence needs assessment. 2013. Available: http://atlas.chimat.org.uk/IAS/profiles/profile?profileId=45&geoTypeId= [Accessed 3 August 2016]

6 Joinson C, Grzeda M, von Gontard A, et al. The association between trajectories of bedwetting and daytime wetting in childhood and incontinence and lower urinary tract symptoms in adolescence. *Archives of Disease in Childhood* 2016*;* 101(S1): A187-188

7 Issenman RM, Filmer RB, Gorski PA. A review of bowel and bladder control development in children: how gastrointestinal and urologic conditions relate to problems in toilet training. *Pediatrics* 1999; 103(6 Pt 2): 1346-52.

8 Bower WF. Self-reported effect of childhood incontinence on quality of life. *Journal of Wound Ostomy & Continence Nursing* 2008; 35(6): 617-21.

9 Landgraf JM, Abidari J, Cilento BG, et al. Coping, commitment, and attitude: quantifying the everyday burden of enuresis on children and their families. *Pediatrics* 2004; 113(2): 334-344.

10 von Gontard A, Baeyens D, Van Hoecke E, et al. Psychological and psychiatric issues in urinary and fecal incontinence. *Journal of Urology* 2011; 185(4): 1432-6.

11 Grzeda M, Heron J, von Gontard A, Joinson C. Effects of urinary incontinence on psychosocial outcomes in adolescence. European Child & Adolescent Psychiatry. In press.

12 Filce HG & LaVergne L. Absenteeism, educational plans, and anxiety among children with incontinence and their parents, *Journal of School Health* 2015; 85(4): 241-250

13 Bower WF. Self-reported effect of childhood incontinence on quality of life, *Journal of Wound, Ostomy, and continence nursing* 2008; 36(6): 617-21.

14 von Gontard A. Enuresis. In Rey JM, eds. *IACAPAP e-Textbook of Child and Adolescent Mental Health.* Geneva: International Association for Child and Adolescent Psychiatry and Allied Professions 2012a

15 von Gontard A. Encopresis. In Rey JM, eds. *IACAPAP e-Textbook of Child and Adolescent Mental Health.* Geneva: International Association for Child and Adolescent Psychiatry and Allied Professions 2012b

16 Abrams P, Anderson KE, Brubaker L, et al. Evaluation and treatment of urinary incontinence, pelvic organ prolapse and faecal incontinence, *3rd International Consultation on Incontinence: Recommendations of the International Scientific Committee.* 2005. Available: http://www.ics.org/publications/ici_3/v2.pdf/summary.pdf [accessed 3 August 2016]

17 Buckley BS, Sanders CD, Kwong JS, et al. Conservative treatment for functional daytime urinary incontinence in children. *Cochrane Database of Systematic Reviews* 2016; 9: 1-11.

18 Maternik M, Krzeminska K, Zurowska A. The management of childhood urinary incontinence. *Pediatric Nephrology* 2015; 30(1): 41-50.

For peer review only - http://bmjopen.bmj.com/site/about/guidelines.xhtml

19 Mulders MM, Cobussen-Boekhorst H, de Gier RP, et al. Urotherapy in children: quantitative measurements of daytime urinary incontinence before and after treatment according to the new definitions of the International Children's Continence Society. *Journal of Pediatric Urology* 2011; 7(2): 213-8.

20 Hagstroem S, Rittig S, Kamperis K, et al.. Timer watch assisted urotherapy in children: a randomized controlled trial. *Journal of Urology* 2010; 184(4): 1482-8.

21 NICE. Bedwetting in under 19s, NICE guidelines. 2010. Available at: https://www.nice.org.uk/guidance/cg111/chapter/Key-priorities-for-implementation [accessed 18 August 2016]

22 Burger RE, Mugie SM, Chase J, et al. Management of functional constipation in children with lower urinary tract symptoms: report from the standardization committee of the International Children's Continence Society. *The Journal of Urology* 2013*;* 190: 29-36.

23 NICE. Constipation in children and young people: diagnosis and management, Clinical Guidelines. 2010. Available at: https://www.nice.org.uk/Guidance/CG99 [accessed 19 Sept 2016]

24 Yeung CK, Sreedhar B, Sihoe JD, et al. Differences in characteristics of nighttime enuresis between children and adolescents: a critical appraisal from a large 338 epidemiological study. *British Journal of Urology International* 2006; 97(5): 1069-1073.

25 Swithinbank LV, Brookes ST, Shepherd AM et al. The natural history of urinary symptoms during adolescence. *British Journal of Urology* 1998;81 Suppl 3:90-3.

26 Hellström AL, Hansson E, Hansson S, et al. Micturition habits and incontinence at age 17 - reinvestigation of a cohort studied at age 7. *British Journal of Urology* 1995; 76: 231-234.

27 Kyrklund K, Taskinen S, Rintala RJ, et al. Lower urinary tract symptoms from 353 childhood to adulthood: a population based study of 594 Finnish individuals 4 to 26 354 years old. *Journal of Urology* 2012; 188(2): 588-93.

28 Taddeo D, Egedy M, Frappier JY. Adherence to treatment in adolescents. *Paediatric Child Health* 2008; 13(1): 19-24.

29 Baeyens D, Lierman, A, Roeyes H, Hoebke P, Vande Walle J. Adherence in children with nocturnal enuresis, *Journal of Pediatric Urology* 2008; 5(2): 105-109.

30 Malterud K, Siersma VD, Guassora AD. Sample size in qualitative interview studies: Guided by information power, *Innovative Methods* 2016; 26(13):1753-1760

31 Carter B. & Ford K. Researching children's health experiences: The place for participatory, child-centred, arts-based approaches, *Research in Nursing and Health* 2013; 36(1): 95-107.

32 Braun V. & Clarke V. Using thematic analysis in psychology. *Qualitative Research in* Psychology 2006; 3(2): 77-101.

33 Tracey SJ. Qualitative quality: Eight "Big-Tent" criteria for excellent qualitative research, *Qualitative Inquiry* 2010; 16(10): 837-851.

34 de Winter M, Baerveldt C, Kooistra J. Enabling children: Participation as a new perspective on child-health promotion. *Child Care Health Development* 2002; 28:109–16.

35 Holtzheimer L, Mohay H, Masters IB. Educating young children about asthma: Comparing the effectiveness of developmentally appropriate asthma education videotape and picture book. *Child Care Health Development* 1998; 24: 85–99.

36 Ginsburg KR, Menapace AS, Slap GB. Factors affecting the decision to seek health care: The voice of adolescents. *Pediatrics* 1997; 100: 922–30.

37 Freed LH, Ellen JM, Irwin CE, et al. Determinants of adolescent satisfaction with health care providers and intentions to keep follow-up appointments. *Journal of Adolescent* Health 1998; 22: 475–9.

For peer review only - http://bmjopen.bmj.com/site/about/guidelines.xhtml

38 Department for Health. Getting the right start: The national service framework for children, young people and maternity services – Standards for hospital services. London: Stationary Office 2008.

39 Deci EL & Ryan RM. Intrinsic Motivation and Self-Determination in Human Behaviour. New York: Plenum Publishing Co. 1985.

40 Deci EL & Ryan RM. Self-determination theory: A macrotheory of human motivation, development, and health. *Psychologie canadienne* 2008; 49(3): 182-185.

41 Mishel MH. Uncertainty in illness. *Image: The Journal of Nursing Scholarship* 1988; 20(4): 225–232.

42 Mishel MH. Reconceptualization of the uncertainty in illness theory. Image: *Journal of Nursing Scholarship* 1990; 22(4): 256-262.

43 Ballif M, Ledergerber B, Battegay, M. Impact of previous virological treatment failures and adherence on the outcome of antitretroviral therapy in 2007, *PLoS ONE 2009;* 4(12): e8275

44 Mann D, Ponieman D, Leventhal H, et al. Predictors of adherence to diabetes medications: The role of disease and medication beliefs. *Journal of Behavioural Medicine* 2009; 32(3): 278-284.

45 Zugelj U, Zupancic M, Komidar L, et al. Self-reported adherence beahviours in adolescent hypertensive patients: the role of illness representations and personality. *Journal of Paediatric Psychology* 2010: 1-12.

46 Leventhal H, Brissette I & Leventhal EA. The common-sense model of self-regulation of health and illness. In: Cameron LD, Leventhal H, eds. The Self-Regulation of Health and Illness behaviour. London: Routledge 2003: 42-65.

47 Wright LJ, Afari N. & Zautra, A. The illness uncertainty concept: a review. *Current Pain and Headache Reports* 2009; 13: 133-138.

48 Williams GC, McGregor HA, Zeldman A, et al. Testing a self-determination theory process model for promoting glycemic control through diabetes self-management. *Health Psychology* 2004; 23(1): 58-66.

49 Hayes SC, Luoma JB, Bond FW, et al. Acceptance and commitment therapy: model, processes and outcomes. *Behaviour Research and Therapy* 2006; 44: 1-25.

50 Leventhal H, Diefenbach M. & Leventhal E. Illness cognition: Using common sense to understand treatment adherence and affect cognition interactions. *Cognitive Therapy and Research* 1992; 16: 143-163.

51 Li L. & Moore D. Acceptance of disability and its correlates. *Journal of Social Psychology* 1998; 138: 13-25.

52 Stuifbergen A, Becker H, Blozis, S, et al. Conceptualization of development of the acceptance of chronic health conditions scale. *Issues in Mental Health Nursing* 2008; 29: 101-114.

53 Karademas EC, Tsagaraki A. & Lambrou N. Illness acceptance, hospitalization stress, and subjective health in a sample of chronic patients admitted to hospital. *Journal of Health Psychology 2009;* 14:1243–1250

For peer review only - http://bmjopen.bmj.com/site/about/guidelines.xhtml

COREQ (COnsolidated criteria for REporting Qualitative research) Checklist

A checklist of items that should be included in reports of qualitative research. You must report the page number in your manuscript where you consider each of the items listed in this checklist. If you have not included this information, either revise your manuscript accordingly before submitting or note N/A.

Topic	Item No.	Guide Questions/Description	Reported on Page No.
**Domain 1: Research team and reflexivity**			
Personal characteristics			
Interviewer/facilitator	1	Which author/s conducted the interview or focus group?	8
Credentials	2	What were the researcher's credentials? E.g. PhD, MD	8
Occupation	3	What was their occupation at the time of the study?	8
Gender	4	Was the researcher male or female?	8
Experience and training	5	What experience or training did the researcher have?	8
Relationship with participants			
Relationship established	6	Was a relationship established prior to study commencement?	7
Participant knowledge of the interviewer	7	What did the participants know about the researcher? e.g. personal goals, reasons for doing the research	7
Interviewer characteristics	8	What characteristics were reported about the inter viewer/facilitator? e.g. Bias, assumptions, reasons and interests in the research topic	8
**Domain 2: Study design**			
Theoretical framework			
Methodological orientation and Theory	9	What methodological orientation was stated to underpin the study? e.g. grounded theory, discourse analysis, ethnography, phenomenology, content analysis	8
Participant selection			
Sampling	10	How were participants selected? e.g. purposive, convenience, consecutive, snowball	7
Method of approach	11	How were participants approached? e.g. face-to-face, telephone, mail, email	7
Sample size	12	How many participants were in the study?	7
Non-participation	13	How many people refused to participate or dropped out? Reasons?	7
Setting			
Setting of data collection	14	Where was the data collected? e.g. home, clinic, workplace	7
Presence of non-participants	15	Was anyone else present besides the participants and researchers?	7
Description of sample	16	What are the important characteristics of the sample? e.g. demographic data, date	9
Data collection			
Interview guide	17	Were questions, prompts, guides provided by the authors? Was it pilot tested?	7-8
Repeat interviews	18	Were repeat inter views carried out? If yes, how many?	7
Audio/visual recording	19	Did the research use audio or visual recording to collect the data?	8
Field notes	20	Were field notes made during and/or after the inter view or focus group?	8
Duration	21	What was the duration of the inter views or focus group?	8
Data saturation	22	Was data saturation discussed?	8
Transcripts returned	23	Were transcripts returned to participants for comment and/or	8

For peer review only

For peer review only - http://bmjopen.bmj.com/site/about/guidelines.xhtml

Topic	Item No.	Guide Questions/Description	Reported on Page No.
		correction?	
**Domain 3: analysis and findings**			
Data analysis			
Number of data coders	24	How many data coders coded the data?	8
Description of the coding tree	25	Did authors provide a description of the coding tree?	n/a
Derivation of themes	26	Were themes identified in advance or derived from the data?	10
Software	27	What software, if applicable, was used to manage the data?	8
Participant checking	28	Did participants provide feedback on the findings?	n/a
Reporting			
Quotations presented	29	Were participant quotations presented to illustrate the themes/findings? Was each quotation identified? e.g. participant number	10-15
Data and findings consistent	30	Was there consistency between the data presented and the findings?	10-15
Clarity of major themes	31	Were major themes clearly presented in the findings?	10-15
Clarity of minor themes	32	Is there a description of diverse cases or discussion of minor themes?	10-15

Developed from: Tong A, Sainsbury P, Craig J. Consolidated criteria for reporting qualitative research (COREQ): a 32-item checklist for interviews and focus groups. *International Journal for Quality in Health Care*. 2007. Volume 19, Number 6: pp. 349 – 357

Once you have completed this checklist, please save a copy and upload it as part of your submission. DO NOT include this checklist as part of the main manuscript document. It must be uploaded as a separate file.

For peer review only

BMJ Open

‘What does that mean?’: A qualitative exploration of the primary and secondary clinical care experiences of young people with continence problems in the UK

Journal:	*BMJ Open*
Manuscript ID	bmjopen-2016-015544.R3
Article Type:	Research
Date Submitted by the Author:	14-Jul-2017
Complete List of Authors:	Whale, Katie; University of Bristol, Centre for Child and Adolescent Health<br>Cramer, Helen; Centre for Academic Primary Care, School of Social and Community Medicine<br>Wright, Anne; Evelina London Children's Hospital<br>Sanders, Caroline ; University of Northern British Columbia<br>Joinson, Carol; University of Bristol, Centre for Child and Adolescent Health; School of Social and Community Medicine, University of Bristol
**Primary Subject Heading**:	Urology
Secondary Subject Heading:	Paediatrics, Qualitative research
Keywords:	Urinary incontinences < UROLOGY, Fecal incontinence, PAEDIATRICS

SCHOLARONE™ Manuscripts

For peer review only

‘What does that mean?’: A qualitative exploration of the primary and secondary clinical care experiences of young people with continence problems in the UK

Whale K[1], Cramer H[2], Wright, A[3], Sanders, C[4] & Joinson C[1]

[1] Centre for Child and Adolescent Health, School of Social and Community Medicine, University of Bristol

[2] Centre for Academic Primary Care, School of Social and Community Medicine, University of Bristol

[3] Evelina London Children’s Hospital, Guy’s and St Thomas’ NHS Foundation Trust, London

[4] University of Northern British Columbia, Canada

Corresponding author

Dr Katie Whale

Centre for Child and Adolescent Health

School of Social and Community Medicine

University of Bristol

Oakfield House

Oakfield Grove

Bristol

BS8 2BN

Email: Katie.whale@bristol.ac.uk

Phone: 0117 3313328

Data sharing statement

Data from the study is classified as controlled access. No participant consent was given to share their interview data, therefore no further data from this study is available.

For peer review only - http://bmjopen.bmj.com/site/about/guidelines.xhtml

For peer review only

Abstract

Objectives: To explore the clinical care experiences of young people with continence problems.

Design: In-depth semi-structured qualitative interviews were conducted by Skype and telephone, with the addition of art-based participatory research techniques. Transcripts were analysed using inductive thematic analysis.

Setting: Primary and secondary care in the UK.

Participants: We interviewed 20 participants (nine females, 11 males) aged 11-20. There were six participants with bedwetting alone, five with daytime wetting alone, five with combined (day and night) wetting, and four with soiling.

Results: We identified four themes: appointment experiences, treatment experiences, engagement with treatment, and internalisation and externalisation of the continence problem. Patient-focused appointments using age-appropriate language were highly desirable. Continuity of care was highlighted as an important aspect of positive clinical experiences, however this was found to be rare with many participants seeing a different person on each visit. Participants had tried a wide range of treatments for their continence problems with varying degrees of success. Relapse and treatment failure were common. Experiencing relapse was distressing and diminished participants' belief in the success of future treatments, and undermined adherence. Participants would be seen to adopt two opposing coping strategies for dealing with their continence problem, internalisation and externalisation.

Conclusion: Incontinence in young people is challenging to manage. Young people may need to try a range of treatments before their symptoms improve. Due to challenges in treatment there is an increased risk of poor adherence. During patient-focused appointments clinicians should work to build rapport with patients and use age-appropriate language. Involving young people in their own care decisions is important. The way in which young people understand their continence problem can influence their coping strategies and adherence to treatment regimes.

For peer review only - http://bmjopen.bmj.com/site/about/guidelines.xhtml

Strengths and limitations of the study

- In-depth qualitative interviews provide a unique insight into the experiences of young people with incontinence
- Due to the sensitive nature of this topic, interviews were combined with an arts-based participatory approach to facilitate greater disclosure and gain a better insight into young people's lives
- Skype and telephone interviews allowed access to participants from a wide geographical area
- Telephone interviews presented challenges in building rapport and body language with the participants, which may have hindered some personal disclosure

This research was funded by a grant from the Medical Research Council (Increasing understanding of risk factors and outcomes associated with continence problems in children and adolescents. MRC reference: MR/L007231/1).

We have read and understood BMJ policy on declaration of interests and declare that we have no competing interests

Author contributions

Katie Whale: Lead researcher, design of qualitative work, conducting interviews, analysis of interview data, writing and preparing manuscript

Helen Cramer: Design of qualitative work, analysis of a sub-set of interviews and input into analysis process, member of the project steering group, revising manuscript critically for important intellectual content

Anne Wright: Recruitment of participants, contribution to interview design, contribution to interpretation of themes, member of the project steering group, revising manuscript critically for important intellectual content

For peer review only - http://bmjopen.bmj.com/site/about/guidelines.xhtml

Caroline Sanders: Contribution to interpretation of themes, revising manuscript critically for important intellectual content

Carol Joinson: Principal investigator, obtaining ethical approval for the study, design of qualitative work, analysis of a sub-set of interviews and input into analysis process, revising manuscript critically for important intellectual content

For peer review only

For peer review only - http://bmjopen.bmj.com/site/about/guidelines.xhtml

INTRODUCTION

Continence problems (daytime wetting, bedwetting, and soiling) [1] are among the most common chronic conditions of childhood. In the UK it is estimated that 900,000 children and young people experience some form of continence problem[2]. This is, however, likely to be an underestimate as the stigma associated with incontinence often prevents individuals from seeking treatment[3, 4]. Epidemiological studies estimate the prevalence of incontinence in 11–20 year olds to be around 2–3% for daytime wetting, 2–2.5% for bedwetting and 1–1.5% for soiling[5, 6]. Incontinence in children and young people has been linked to high rates of emotional distress, poor self-image, relationship problems and unhappiness at school[7-12]. Poor management of continence problems has an adverse impact on quality of life and self-esteem during childhood and adolescence[13].

A minority of children and young people have an organic (neurological, structural or anatomic) cause for their incontinence, but the vast majority of paediatric continence problems are functional (no underlying organic cause)[14, 15, 1]. Uncertainty surrounding individual responses to treatments for functional incontinence and varying levels of access to paediatric continence services are major factors affecting patients with incontinence[2]. Treatment for functional incontinence is varied and there is often no singularly effective treatment. As a consequence, patients may need to try a series of different approaches to manage or improve their incontinence symptoms[16, 17]. The first-line treatment for daytime wetting is often urotherapy, involving a structured regime of toileting and fluid intake, sometimes combined with medication[18-20]. Initial treatments for bedwetting include the bedwetting alarm and/or medication (e.g. desmopressin)[16, 21, 14]. Constipation is a common cause of soiling and can exacerbate urinary incontinence due to pressure on the bladder. The first stage of treatment for soiling usually involves laxatives or stool softeners in addition to a toileting programme and, in some cases, suppositories[22, 15, 23].

Although many children experience a natural resolution of their incontinence with age, there is evidence that those with severe incontinence are at risk of their problems persisting into adolescence [6, 24-27]. Often when curative treatment fails there is a refocusing on symptom management as the child continues to mature and enters into adolescence. Adolescence can be a particularly challenging time for the management of chronic health

For peer review only - http://bmjopen.bmj.com/site/about/guidelines.xhtml

problems and is frequently associated with lower levels of treatment adherence[28]. Due to the challenges of treatment, young people with persistent continence problems may see minimal improvements and may need to follow treatment regimes for extended periods before seeing any changes. Previous work has shown that adolescents often struggle to adhere to treatments that show few immediate benefits[28,29].

Little research has been published on the primary and secondary care experiences of young people with continence problems, especially with regard to their experiences of treatment and management of their continence problems. The overall aim of the project was to explore the impact of continence problems on young people and the specific focus of this paper is on their experiences of primary and secondary care.

For peer review only

METHODS

Participants were recruited through five secondary care paediatric continence clinics (four in England and one in Scotland), and through an advertisement on a paediatric continence charity website (The Children's Bowel and Bladder Charity, www.eric.org.uk).
Eligible participants were between 11 and 20 years, currently experiencing continence problems (bedwetting, daytime wetting, or soiling) or who previously experienced these problems after the age of 10, and able to speak and understand English.

Participants attending paediatric continence clinics were given a study information pack by their clinician. Those recruited through the advertisement were sent an information pack by the research team. Ethical approval was given by the National Research Ethics Service Committee South West - Central Bristol (14/SW/0059). The researcher contacted all interested participants by phone to describe the study, the motivation for the work, and answer any questions. All participants were unknown to the researcher prior to the commencement of the study. In total 45 potential participants were identified (36 through clinics and 9 through ERIC). 25 participants were not interviewed for the following reasons: 12 unable to contact, 5 not interested in taking part, 8 withdrawn.

In-depth semi-structured interviews were conducted with 20 young people between February 2015 and January 2016 (see table 1 for participant characteristics). Recruitment and final sample size were guided by the concept of 'information power'[30]. Given the specificity of the sample, broad study aim, and strong interview dialogue, a sample size of 20 participants was considered appropriate.

Participants were given the option to be interviewed by telephone, Skype, or face to face (for participants within a 40 mile radius of Bristol). Among the participants, 11 were interviewed by Skype and nine by telephone. No participants were interviewed face to face (those given this option preferred to be interviewed by Skype or telephone). No repeat interviews were conducted. Four participants chose to have their parent/guardian present during the interview, seven were in a communal area of their house, and nine chose to conduct the interview alone in a private room.

For peer review only - http://bmjopen.bmj.com/site/about/guidelines.xhtml

A topic guide was developed based on previous literature on the treatment, management and impact of continence problems in children and young people. The topic guide covered issues such as attending appointments, treatment experiences, school or work, and thoughts and feelings about their continence problem. Due to the exploratory nature of the study the interviews were semi-structured. The topic guide was used as a starting point for discussion, with flexibility to discuss novel areas introduced by the participants.

An arts-based participatory approach was used in the interviews. This approach is considered appropriate for children and young people since it provides additional narratives through which personal experiences can be explored[31]. A participant activity pack was developed for use prior to and during the interviews containing a graphic representation of each possible topic area, allowing the participants to write or draw their thoughts in advance, if they wished to. Participants were sent the pack in advance of their interview and were given a verbal explanation of how it could be used during the initial phone call.

All participants provided written informed consent, for ages 16 and above, or parental written consent and child assent for those below 16 years. Interviews were conducted by one female researcher with extensive experience of qualitative work (KW: DHealthPsy, Senior Research Associate) and lasted between 34 and 99 minutes (mean 65 minutes). The researcher made detailed field notes after each interview, including details about the environment, participant demeanour, and personal reflections. Data were recorded using an encrypted audio recorder. Data collection and analysis were conducted in parallel after completion of the first five interviews. Early analysis was used to refine the topic guide and to further explore emerging areas of interest. Interviews were audio recorded, fully transcribed, and imported into the software package NVivo10. Inductive thematic analysis was carried out following guidelines of Braun and Clarke[32]. Following completion of the first five interviews, each transcript was read and the data were free-coded across all transcripts. A selection of three transcripts were also independently free coded by the study team (CJ and HC). Codes were discussed and compared with all members of the team in order to further refine coding and to maximise rigor[33]. An initial set of agreed codes were set up within the NVivo10 database and any new codes identified from further interviews were

For peer review only - http://bmjopen.bmj.com/site/about/guidelines.xhtml

discussed within the team and added to the coding framework. A strong theme was identified from the data on young people's experiences of clinical care.

Sample description

In total, 20 interviews were carried out with children and young people aged 11-20 years. 17 were recruited through paediatric continence clinics and three through the ERIC advertisement. Three had organic incontinence and 17 had non-organic incontinence. Table 1 provides a full overview of participant characteristics.

Table 1: Participant characteristics

Participant ID no.	Gender	Age group*	Continence problem	Method of interview
1	M	11-13	Night wetting	Skype
2	M	14-16	Day and night wetting	Telephone
3	F	17-19	Daytime wetting	Skype
4	F	14-16	Daytime wetting	Skype
5	F	11-13	Day and night wetting	Skype
6	M	14-16	Day and night wetting	Skype
7	M	11-13	Night wetting	Telephone
8	M	11-13	Night wetting	Telephone
9	M	11-13	Day and night wetting	Telephone
10	F	14-16	Night wetting	Skype
11	M	14-16	Daytime wetting	Skype
12	M	17-19	Night wetting	Telephone
13	F	11-13	Soiling	Skype
14	F	11-13	Soiling	Skype
15	F	14-16	Daytime wetting	Skype
16	F	14-16	Daytime wetting	Telephone
17	M	11-13	Soiling	Telephone
18	F	17-19	Night wetting	Telephone
19	M	11-13	Soiling	Telephone
20	M	14-16	Day and night wetting	Skype

*For confidentiality reasons, participant age is given in group

For peer review only

For peer review only - http://bmjopen.bmj.com/site/about/guidelines.xhtml

RESULTS

Themes

Three themes directly linking to clinical care experiences were identified from the data: *appointment experiences*, *treatment experiences*, and *engagement with treatment*. A fourth theme was identified from data that was not directly linked to clinical experiences but was felt to have important implications for self-management and adherence; *internalisation and externalisation of the continence problem*.

Appointment experiences: what makes a good appointment?

Participants in the study had seen a range of health professionals during their treatment journey including general practitioners (GPs), community and school nurses, paediatricians, urologists, and paediatric continence specialists. Participants' first contact with health services was most commonly with a GP or a community or school nurse. Participants recruited through the paediatric continence clinics had the experience of attending a specialist service, whilst those recruited through the online advertisement had primarily seen their GP, or a small number had seen a general paediatrician.

For all participants, the most important factor that influenced their appointment experiences was the communication style used by the clinician, specifically, whether the clinician used patient-centred communication and age-appropriate language. Participants expressed a strong desire for the clinician to include them in the conversation and talk to them directly (rather than to their parent), and to use language that they could understand.

> *"The language they used, and everything, made it easier. It made it kind of child friendly I suppose, which is what they should be doing." – P12, male, aged 19, night wetting*

When health professionals failed to do this, participants talked about feeling confused and annoyed.

> *"I always had to ask my mum 'what does that mean? […] he didn't even explain what it was and even my mum didn't really know what it was half the time, because they say things that they think that you'll know, but you don't know. That's frustrating*

For peer review only - http://bmjopen.bmj.com/site/about/guidelines.xhtml

because it's like, I don't know what that means and they're not telling me what that means". – P14, female, age 12, soiling

Due to the sensitive nature of continence problems, appointments often involved questions and discussions about very personal and potentially embarrassing issues. Therefore, building trust and rapport with the clinician was especially important for young people. Continuity of care was highlighted as a factor that helped build rapport and was highly desirable. Participants who reported seeing the same clinician at each appointment described how this helped them to feel more comfortable.

"After my second visit I got to know her a little better and I felt more comfortable every time I went". – P9, male, age 11, day and night wetting

Continuity of care was more likely when participants had been referred to a specialist continence clinic, but was not always guaranteed. More than half of the participants described seeing a different clinician on each visit with no explanation as to why. Participants said that new clinicians were not familiar with their case history, or gave conflicting advice to previous clinicians.

"when we're at the hospital it's always a new person and they always say a different thing to what the other doctor said." – P1, male, age 12, night wetting

Appointments often centred on developing treatment plans to be implemented independently at home by the young person and/or parent. Participants expressed a strong desire to be included in the decision-making process, through being given a rationale for each treatment option and a degree of choice in how to use the treatment plan at home. When health professionals engaged in shared decision-making approaches, participants reported positive appointment experiences.

"I like to know what to do and why I should do it". – P8, male, age 11, night wetting

"He's just really warm and stuff and he just says 'How are you feeling? Do you want to do that? Do you not want to do that?'". – P14, female, age 12, soiling

For peer review only - http://bmjopen.bmj.com/site/about/guidelines.xhtml

Participants described wanting to be treated as an intelligent person and not wanting to be patronised by their clinician. Being seen as an expert in relation to themselves and their body was important.

> *"I like them to treat me as if I'm the person in question, because I'm not stupid, I know I'm not brilliant at medicine or anything, but I can do my research. I know about the medics of it. I know about the treatments that I had, and I know about me, more than anyone". – P2, male, age 14, day and night wetting*

Treatment experiences: the frustration of no magic bullets

Participants often recalled trying a wide range of treatments including urotherapy, dietary changes, medication(s), bedwetting alarms, Botox injections into the bladder, laxatives, suppositories, TENS machine, and hypnotherapy. Almost all participants had tried more than one treatment and had experienced some form of relapse or complete treatment failure. Participants explained that with each failed treatment or relapse, their belief in the success of subsequent treatments diminished.

> *"For the first four months, it was fine…it was a lot better. Then it started to get worse again, and by six or seven months, it was actually worse than it was before I had the [Botox] operation. We went back to do a second one…it was fine for six months. Then it got worse again, and then they called us back to have another one, and we said no. By that point, I was kind of sick of it, and I knew that it wasn't going to work". – P2, male, age 14, day and night wetting*

Belief in treatments seemed to be made much more difficult because of the uncertainty of the cause of young people's continence problems. A minority of participants had been given a medical explanation for their incontinence and reported that this was helpful with respect to the treatments then offered.

> "*I think it made me more comfortable, knowing it was actually a problem and it wasn't just my mum being frantic or something*". – P4, female, age 14, daytime wetting

The majority of participants, however, were given no medical explanation for their incontinence. Participants said that they found this frustrating and confusing.

For peer review only - http://bmjopen.bmj.com/site/about/guidelines.xhtml

"I really struggled, just thinking, 'Why has it become a problem for me?' Because there was not any reason". – P3, female, age 18, daytime wetting

Not being able to match suggested treatments to an underlying cause of the incontinence was also challenging for participants to understand. Other participants had been told there was nothing wrong with them.

"Him saying there's nothing wrong with me, he made me feel quite vulnerable […] it really made me feel bad because we were told that he is the man to see…if he can't do anything about it, like who can?". – P11, male, age 14, daytime wetting

Participants talked extensively about their emotional reactions to treatments saying that the lack of explanation, unpredictability of treatment success combined with experiences of relapse, and treatment failure, led them to feel upset and cynical about the chances of resolution. Many described giving up hope that their problem would ever go away.

"Some of it's obviously a little bit upsetting because I've had so many different medications and trying new things and it hasn't really worked yet, so my hope of it going to getting smaller and smaller". – P16, female, age 15, daytime wetting

For older participants, who had often been undergoing treatment for several years, feelings of pessimism were particularly common.

"For quite a lot of the time the, I felt really really pessimistic about this problem, just like there is absolutely no hope that it's ever going to be better and I'm going to be stuck with this problem forever. Clearly I don't want that to happen, so that really upsets me about it". – P3, female, age 18, daytime wetting

Engagement with treatment: the effects of disappointing results

Participants reported varying levels of engagement with their treatment. Experiencing relapse or treatment failure led many participants to disengage from their treatment, with some admitting that they avoided taking their medication or lied about this to their parents.

"Instead of taking the tablets, I think mum thought I was doing it, say, once a week or something, I was [throwing them away] every day and having problems every day because of that".- P4, female, age 14, daytime wetting

For peer review only - http://bmjopen.bmj.com/site/about/guidelines.xhtml

One participant talked about feeling so frustrated with the lack of improvement in her symptoms that she decided no treatment was going to work and there was no point trying anything new.

> *"I think it was because I'd been on them [the medication] for so long, I didn't feel like they were doing anything. I've never actually tried it [the new medication]. I've never actually properly tried it […] I just decided that it wouldn't work either". – P4, female, age 14, daytime wetting*

Another participant described disengaging from treatment not due to lack of belief in its efficacy, but as an effort to regain her independence.

> *"I think there was a period when I was in year nine [aged 13-14] where I didn't take my medication very well and it just got worse. […] I was just like 'I can do it on my own, I'm an independent woman, I can do what I want". – P15, female, age 14, daytime wetting*

Internalisation and externalisation of the continence problem

Through discussion with participants about their beliefs and attitudes towards their continence problem, two contrasting coping mechanisms were identified; internalisation and externalisation of the continence problem. Participants who internalised their continence problem integrated it into their self-identity. They viewed the problem as a part of their lives and themselves that needed to be actively managed, but not as something that defined or controlled them. Participants who demonstrated internalisation talked about a process of accepting that they had a continence problem, and the need to actively manage it. Taking ownership of their continence problem and embracing it because it made them 'different' and 'unique' were common lines of thought. Others talked about how this attitude helped them to cope with worries about other people finding out about their problem.

> *"I kind of realised that, actually, it's something I can't change, and it's a part of me, so I shouldn't be scared of people knowing. It's not something I've done wrong" – P2, male, age 14, daytime and night wetting*

For peer review only - http://bmjopen.bmj.com/site/about/guidelines.xhtml

In contrast, those who externalised their continence problem saw it as something separate to themselves and their identity, and as an intruder into their lives. Participants who demonstrated externalisation often avoided thinking about their continence problem and talked about distancing themselves from their problem and rejecting it from their self-identity.

> *"…it's just frustrating also because you know what's going to happen. So you kind of always, that's always at the back of your mind. So I do then just try and avoid it [thinking about it] if I can" – P12, male, age 19, night wetting*

In order to deal with stressful social situations, such as sleepovers or school trips, participants said they tried to convince themselves it would just be fine, even if they knew this was highly unlikely.

> *"I think when you have this issue you're trying to put aside, you know the problems but you're trying to get it out of your head, so you will do all you can to convince yourself that you'll be fine even if you won't be". – P11, male, age 14, night wetting*

For peer review only - http://bmjopen.bmj.com/site/about/guidelines.xhtml

DISCUSSION

Appointment experiences

Through interviews with young people with continence problems, we find that positive treatment experiences are characterised by seeing the same clinician at each appointment and by the use of age-appropriate communication. Building a relationship with the same clinician was desirable as it facilitated greater trust and rapport and supported young people to feel more comfortable disclosing highly personal information. Participants expressed a strong desire to be given a full explanation of their continence problem, appropriate to their age and level of understanding. This finding is consistent with studies of clinical experiences of young people with other chronic health problems[34-37]. Young people in this study wanted to be involved in making decisions about their treatment options, rather than the traditional focus on parent-doctor communication in paediatric consultations. Aligned to this, it is now recommended that children and young people should be the primary focus of their own appointments[38, 16]. There is evidence that this facilitates better communication and patient understanding, supports patient autonomy and competence, and promotes successful behaviour change and illness management[39, 40]. Shared decision making between clinician and patient could aid treatment engagement and autonomy in young people and provide them with skills and confidence to transition into adult care.

Factors affecting treatment engagement

The majority of participants in this study had functional incontinence (n= 17). In the absence of an underlying organic cause of the incontinence, clinicians were often unable to give specific guidance on treatments and prognosis, creating significant illness uncertainty for the participants[41, 42]. This presents challenges since participants often had to try a range of treatments, with varying success[1]. Treatment failures and relapses were common and undermined participants' beliefs in future treatment success and the controllability of their continence problem.

Illness uncertainty and lack belief in the controllability of a health problem are significant barriers to adherence and engagement, because individuals are less likely to follow a

For peer review only - http://bmjopen.bmj.com/site/about/guidelines.xhtml

treatment regime they do not believe will work[43, 44]. Models of health behaviour change consistently highlight the perceived controllability of a health problem as a key factor in predicting health behaviours and treatment adherence[45-46]. Additionally, illness uncertainty has been linked to maladaptive coping, increased psychological distress, depression, and reduced quality of life[47]. When illness uncertainty is an issue, it is crucial that clinicians work to positively engage patients and support them in long-term self-management in order to minimise the impact of their health problem on daily life. Successful self-management also promotes great individual autonomy and can help patients to feel more in control of their bodies[48]. Young people with functional incontinence could also benefit from psychological support and interventions to manage uncertainty and reduce anxiety, such as acceptance commitment therapy[49]. Finding a balance between giving patients hope and being realistic about treatment success is vital in managing patient expectations, and could help to mitigate against negative treatment experiences. By increasing understanding of how treatment failures and relapses affect patients, health professionals can identify potential periods of high-risk non-adherence and take steps to manage this.

Internalisation and externalisation of the problem

Amongst the participants in our sample there was a dichotomy between those who demonstrated internalisation and externalisation of their continence problem. Those who internalised their continence problem had more successfully incorporated this into their self-identity. They recognised it as an ongoing part of their lives, but did not feel restrained or defined by it. In contrast, those who externalised their problem saw it as an external factor that intruded into their life and avoidance coping was common in this group.

Although participants did not directly link these coping strategies to their clinical experiences, the way in which they view their health condition does have significant implications for self-management and adherence to treatment. By viewing continence problems as external to the self, this undermines beliefs in the controllability of the condition, which are linked to treatment adherence[45, 46, 50]. If an individual engages in avoidance coping, they are likely to avoid any stimulus that reminds them of their continence problem, including treatment regimes. Non-adherence increases the risk of treatment failure or relapses, which may further reinforce the belief that the condition is

For peer review only - http://bmjopen.bmj.com/site/about/guidelines.xhtml

For peer review only

uncontrollable, creating a self-perpetuating cycle of disengagement and treatment failure. In contrast, acceptance of a chronic health problem and integration of illness experiences where the condition is viewed as only one characteristic of an individual's life are linked to a greater sense of personal control and proactive management behaviours[51-53].

Whilst within our sample internalisation and externalisation are seen as two different groups, it is likely that within the wider population these responses fall along a continuum. Additionally, individuals may be likely to switch between the two attitudes depending on their experiences. Experiencing relapse or feeling overwhelmed by the condition may promote more externalising attitudes in order to cope. Alternatively, successful treatment and management symptoms may support greater internalisation. Without more exploration of these issues, the exact relationships and mechanism at work are not clear, and further work is needed to better understand this.

LIMITATIONS

Only a small number of participants with soiling were recruited into this study. Young people with soiling problems reported some different experiences to those with urinary incontinence, suggesting that further exploration is needed. Clinicians involved in recruiting to the study reported that young people with soiling were generally less engaged during appointments and would often not admit to having a problem. This led to a problem recruiting these individuals. The participants with soiling either had a medical explanation for their problem (n=1), or were experiencing significant improvements in their symptoms (n=3). Another limitation of the sample was that all the participants in this study were receiving medical help for their continence problem and the majority were seeing specialists. Young people who have not received specialist help for their continence problems are likely to have different experiences to the participants in this study. This study could not explore cultural differences in young people's experiences of continence problems since all but one of the participants identified as white British. In addition, as all participants were from the UK, we do not know if the same issues are experiences in different countries. Socioeconomic background could also impact on experiences of young people with continence problems, but this data was not collected in the current study. As interviews were conducted by Skype and telephone it is possible that some nuances of the

For peer review only - http://bmjopen.bmj.com/site/about/guidelines.xhtml

participants' responses and body language may have been missed. Although this does not change the results, face to face interviews may have yielded an additional level of interpretation and insight.

CONCLUSIONS AND FUTURE DIRECTIONS

Incontinence is challenging to treat and manage, particularly when there is no organic cause. Long-term self-management and helping individuals regain a sense of control is key in minimising the impact of incontinence on daily life. Ensuring positive appointment experiences and engaging young people in their own healthcare is vital. A focus on shared decision-making and ensuring continuity of care are both highly important and facilitate greater engagement with treatment. Clinicians should use age-appropriate language to ensure patient understanding and work to build rapport in order to promote trust and disclosure. Relapses or treatment failure are common and can have a negative emotional impact and undermine beliefs in future treatment success. Assessment of coping styles may a beneficial addition to clinical assessment. For patients who are disengaged or have low treatment adherence, providing psychological support to promote acceptance may be an important first step in successful long-term self-management. These results demonstrate that the same challenges are experienced across the age range and for both organic and non-organic continence problems; this reinforces the importance of addressing these issues.

For peer review only

For peer review only - http://bmjopen.bmj.com/site/about/guidelines.xhtml

REFERENCES

1 Neveus T, von Gontard A, Hoebeke P, et al. The standardisation of terminology of lower urinary tract function in children and adolescents: Report from the standardisation committee of the International Children's Continence Society. *The Journal of Urology,* 2006;176: 314-324

2 Public Health England. Continence Needs Assessment module. 2013. Available: http://atlas.chimat.org.uk/IAS/profiles/needsassessments [Accessed 27th April 2017]

3 NICE. Assumptions used in estimating a population benchmark. 2015. Available: https://www.nice.org.uk/proxy/?sourceurl=http://www.nice.org.uk/usingguidance/commissioningguides/biologicaltherapies/assumptionsusedinestimatingapopulationbenchmark.jsp [Accessed 3 August 2016]

4 NICE. Excellence in continence care. 2015. Available: https://www.england.nhs.uk/commissioning/wp-content/uploads/sites/12/2015/11/EICC-guidance-final-document.pdf [accessed 31 August 2016)

5 Public Health England. Continence needs assessment. 2013. Available: http://atlas.chimat.org.uk/IAS/profiles/profile?profileId=45&geoTypeId= [Accessed 3 August 2016]

6 Joinson C, Grzeda M, von Gontard A, et al. The association between trajectories of bedwetting and daytime wetting in childhood and incontinence and lower urinary tract symptoms in adolescence. *Archives of Disease in Childhood* 2016*;* 101(S1): A187-188

7 Issenman RM, Filmer RB, Gorski PA. A review of bowel and bladder control development in children: how gastrointestinal and urologic conditions relate to problems in toilet training. *Pediatrics* 1999; 103(6 Pt 2): 1346-52.

8 Bower WF. Self-reported effect of childhood incontinence on quality of life. *Journal of Wound Ostomy & Continence Nursing* 2008; 35(6): 617-21.

9 Landgraf JM, Abidari J, Cilento BG, et al. Coping, commitment, and attitude: quantifying the everyday burden of enuresis on children and their families. *Pediatrics* 2004; 113(2): 334-344.

10 von Gontard A, Baeyens D, Van Hoecke E, et al. Psychological and psychiatric issues in urinary and fecal incontinence. *Journal of Urology* 2011; 185(4): 1432-6.

11 Grzeda M, Heron J, von Gontard A, Joinson C. Effects of urinary incontinence on psychosocial outcomes in adolescence. European Child & Adolescent Psychiatry. In press.

12 Filce HG & LaVergne L. Absenteeism, educational plans, and anxiety among children with incontinence and their parents, *Journal of School Health* 2015; 85(4): 241-250

13 Bower WF. Self-reported effect of childhood incontinence on quality of life, *Journal of Wound, Ostomy, and continence nursing* 2008; 36(6): 617-21.

14 von Gontard A. Enuresis. In Rey JM, eds. *IACAPAP e-Textbook of Child and Adolescent Mental Health.* Geneva: International Association for Child and Adolescent Psychiatry and Allied Professions 2012a

15 von Gontard A. Encopresis. In Rey JM, eds. *IACAPAP e-Textbook of Child and Adolescent Mental Health.* Geneva: International Association for Child and Adolescent Psychiatry and Allied Professions 2012b

16 Abrams P, Anderson KE, Brubaker L, et al. Evaluation and treatment of urinary incontinence, pelvic organ prolapse and faecal incontinence, *3rd International Consultation on Incontinence: Recommendations of the International Scientific Committee.* 2005. Available: http://www.ics.org/publications/ici_3/v2.pdf/summary.pdf [accessed 3 August 2016]

17 Buckley BS, Sanders CD, Kwong JS, et al. Conservative treatment for functional daytime urinary incontinence in children. *Cochrane Database of Systematic Reviews* 2016; 9: 1-11.

18 Maternik M, Krzeminska K, Zurowska A. The management of childhood urinary incontinence. *Pediatric Nephrology* 2015; 30(1): 41-50.

For peer review only - http://bmjopen.bmj.com/site/about/guidelines.xhtml

19 Mulders MM, Cobussen-Boekhorst H, de Gier RP, et al. Urotherapy in children: quantitative measurements of daytime urinary incontinence before and after treatment according to the new definitions of the International Children's Continence Society. *Journal of Pediatric Urology* 2011; 7(2): 213-8.

20 Hagstroem S, Rittig S, Kamperis K, et al.. Timer watch assisted urotherapy in children: a randomized controlled trial. *Journal of Urology* 2010; 184(4): 1482-8.

21 NICE. Bedwetting in under 19s, NICE guidelines. 2010. Available at: https://www.nice.org.uk/guidance/cg111/chapter/Key-priorities-for-implementation [accessed 18 August 2016]

22 Burger RE, Mugie SM, Chase J, et al. Management of functional constipation in children with lower urinary tract symptoms: report from the standardization committee of the International Children's Continence Society. *The Journal of Urology* 2013*;* 190: 29-36.

23 NICE. Constipation in children and young people: diagnosis and management, Clinical Guidelines. 2010. Available at: https://www.nice.org.uk/Guidance/CG99 [accessed 19 Sept 2016]

24 Yeung CK, Sreedhar B, Sihoe JD, et al. Differences in characteristics of nighttime enuresis between children and adolescents: a critical appraisal from a large 338 epidemiological study. *British Journal of Urology International* 2006; 97(5): 1069-1073.

25 Swithinbank LV, Brookes ST, Shepherd AM et al. The natural history of urinary symptoms during adolescence. *British Journal of Urology* 1998;81 Suppl 3:90-3.

26 Hellström AL, Hansson E, Hansson S, et al. Micturition habits and incontinence at age 17 - reinvestigation of a cohort studied at age 7. *British Journal of Urology* 1995; 76: 231-234.

27 Kyrklund K, Taskinen S, Rintala RJ, et al. Lower urinary tract symptoms from 353 childhood to adulthood: a population based study of 594 Finnish individuals 4 to 26 354 years old. *Journal of Urology* 2012; 188(2): 588-93.

28 Taddeo D, Egedy M, Frappier JY. Adherence to treatment in adolescents. *Paediatric Child Health* 2008; 13(1): 19-24.

29 Baeyens D, Lierman, A, Roeyes H, Hoebke P, Vande Walle J. Adherence in children with nocturnal enuresis, *Journal of Pediatric Urology* 2008; 5(2): 105-109.

30 Malterud K, Siersma VD, Guassora AD. Sample size in qualitative interview studies: Guided by information power, *Innovative Methods* 2016; 26(13):1753-1760

31 Carter B. & Ford K. Researching children's health experiences: The place for participatory, child-centred, arts-based approaches, *Research in Nursing and Health* 2013; 36(1): 95-107.

32 Braun V. & Clarke V. Using thematic analysis in psychology. *Qualitative Research in* Psychology 2006; 3(2): 77-101.

33 Tracey SJ. Qualitative quality: Eight "Big-Tent" criteria for excellent qualitative research, *Qualitative Inquiry* 2010; 16(10): 837-851.

34 de Winter M, Baerveldt C, Kooistra J. Enabling children: Participation as a new perspective on child-health promotion. *Child Care Health Development* 2002; 28:109–16.

35 Holtzheimer L, Mohay H, Masters IB. Educating young children about asthma: Comparing the effectiveness of developmentally appropriate asthma education videotape and picture book. *Child Care Health Development* 1998; 24: 85–99.

36 Ginsburg KR, Menapace AS, Slap GB. Factors affecting the decision to seek health care: The voice of adolescents. *Pediatrics* 1997; 100: 922–30.

37 Freed LH, Ellen JM, Irwin CE, et al. Determinants of adolescent satisfaction with health care providers and intentions to keep follow-up appointments. *Journal of Adolescent* Health 1998; 22: 475–9.

For peer review only - http://bmjopen.bmj.com/site/about/guidelines.xhtml

38 Department for Health. Getting the right start: The national service framework for children, young people and maternity services – Standards for hospital services. London: Stationary Office 2008.

39 Deci EL & Ryan RM. Intrinsic Motivation and Self-Determination in Human Behaviour. New York: Plenum Publishing Co. 1985.

40 Deci EL & Ryan RM. Self-determination theory: A macrotheory of human motivation, development, and health. *Psychologie canadienne* 2008; 49(3): 182-185.

41 Mishel MH. Uncertainty in illness. *Image: The Journal of Nursing Scholarship* 1988; 20(4): 225–232.

42 Mishel MH. Reconceptualization of the uncertainty in illness theory. Image: *Journal of Nursing Scholarship* 1990; 22(4): 256-262.

43 Ballif M, Ledergerber B, Battegay, M. Impact of previous virological treatment failures and adherence on the outcome of antitretroviral therapy in 2007, *PLoS ONE 2009;* 4(12): e8275

44 Mann D, Ponieman D, Leventhal H, et al. Predictors of adherence to diabetes medications: The role of disease and medication beliefs. *Journal of Behavioural Medicine* 2009; 32(3): 278-284.

45 Zugelj U, Zupancic M, Komidar L, et al. Self-reported adherence beahviours in adolescent hypertensive patients: the role of illness representations and personality. *Journal of Paediatric Psychology* 2010: 1-12.

46 Leventhal H, Brissette I & Leventhal EA. The common-sense model of self-regulation of health and illness. In: Cameron LD, Leventhal H, eds. The Self-Regulation of Health and Illness behaviour. London: Routledge 2003: 42-65.

47 Wright LJ, Afari N. & Zautra, A. The illness uncertainty concept: a review. *Current Pain and Headache Reports* 2009; 13: 133-138.

48 Williams GC, McGregor HA, Zeldman A, et al. Testing a self-determination theory process model for promoting glycemic control through diabetes self-management. *Health Psychology* 2004; 23(1): 58-66.

49 Hayes SC, Luoma JB, Bond FW, et al. Acceptance and commitment therapy: model, processes and outcomes. *Behaviour Research and Therapy* 2006; 44: 1-25.

50 Leventhal H, Diefenbach M. & Leventhal E. Illness cognition: Using common sense to understand treatment adherence and affect cognition interactions. *Cognitive Therapy and Research* 1992; 16: 143-163.

51 Li L. & Moore D. Acceptance of disability and its correlates. *Journal of Social Psychology* 1998; 138: 13-25.

52 Stuifbergen A, Becker H, Blozis, S, et al. Conceptualization of development of the acceptance of chronic health conditions scale. *Issues in Mental Health Nursing* 2008; 29: 101-114.

53 Karademas EC, Tsagaraki A. & Lambrou N. Illness acceptance, hospitalization stress, and subjective health in a sample of chronic patients admitted to hospital. *Journal of Health Psychology 2009;* 14:1243–1250

For peer review only

COREQ (COnsolidated criteria for REporting Qualitative research) Checklist

A checklist of items that should be included in reports of qualitative research. You must report the page number in your manuscript where you consider each of the items listed in this checklist. If you have not included this information, either revise your manuscript accordingly before submitting or note N/A.

Topic	Item No.	Guide Questions/Description	Reported on Page No.
**Domain 1: Research team and reflexivity**			
Personal characteristics			
Interviewer/facilitator	1	Which author/s conducted the interview or focus group?	8
Credentials	2	What were the researcher's credentials? E.g. PhD, MD	8
Occupation	3	What was their occupation at the time of the study?	8
Gender	4	Was the researcher male or female?	8
Experience and training	5	What experience or training did the researcher have?	8
Relationship with participants			
Relationship established	6	Was a relationship established prior to study commencement?	7
Participant knowledge of the interviewer	7	What did the participants know about the researcher? e.g. personal goals, reasons for doing the research	7
Interviewer characteristics	8	What characteristics were reported about the inter viewer/facilitator? e.g. Bias, assumptions, reasons and interests in the research topic	8
**Domain 2: Study design**			
Theoretical framework			
Methodological orientation and Theory	9	What methodological orientation was stated to underpin the study? e.g. grounded theory, discourse analysis, ethnography, phenomenology, content analysis	8
Participant selection			
Sampling	10	How were participants selected? e.g. purposive, convenience, consecutive, snowball	7
Method of approach	11	How were participants approached? e.g. face-to-face, telephone, mail, email	7
Sample size	12	How many participants were in the study?	7
Non-participation	13	How many people refused to participate or dropped out? Reasons?	7
Setting			
Setting of data collection	14	Where was the data collected? e.g. home, clinic, workplace	7
Presence of non-participants	15	Was anyone else present besides the participants and researchers?	7
Description of sample	16	What are the important characteristics of the sample? e.g. demographic data, date	9
Data collection			
Interview guide	17	Were questions, prompts, guides provided by the authors? Was it pilot tested?	7-8
Repeat interviews	18	Were repeat inter views carried out? If yes, how many?	7
Audio/visual recording	19	Did the research use audio or visual recording to collect the data?	8
Field notes	20	Were field notes made during and/or after the inter view or focus group?	8
Duration	21	What was the duration of the inter views or focus group?	8
Data saturation	22	Was data saturation discussed?	8
Transcripts returned	23	Were transcripts returned to participants for comment and/or	8

For peer review only

Topic	Item No.	Guide Questions/Description	Reported on Page No.
		correction?	
**Domain 3: analysis and findings**			
Data analysis			
Number of data coders	24	How many data coders coded the data?	8
Description of the coding tree	25	Did authors provide a description of the coding tree?	n/a
Derivation of themes	26	Were themes identified in advance or derived from the data?	10
Software	27	What software, if applicable, was used to manage the data?	8
Participant checking	28	Did participants provide feedback on the findings?	n/a
Reporting			
Quotations presented	29	Were participant quotations presented to illustrate the themes/findings? Was each quotation identified? e.g. participant number	10-15
Data and findings consistent	30	Was there consistency between the data presented and the findings?	10-15
Clarity of major themes	31	Were major themes clearly presented in the findings?	10-15
Clarity of minor themes	32	Is there a description of diverse cases or discussion of minor themes?	10-15

Developed from: Tong A, Sainsbury P, Craig J. Consolidated criteria for reporting qualitative research (COREQ): a 32-item checklist for interviews and focus groups. *International Journal for Quality in Health Care*. 2007. Volume 19, Number 6: pp. 349 – 357

Once you have completed this checklist, please save a copy and upload it as part of your submission. DO NOT include this checklist as part of the main manuscript document. It must be uploaded as a separate file.

For peer review only
